# Preoperative/Neoadjuvant Therapy in Pancreatic Cancer: A Systematic Review and Meta-analysis of Response and Resection Percentages

**DOI:** 10.1371/journal.pmed.1000267

**Published:** 2010-04-20

**Authors:** Sonja Gillen, Tibor Schuster, Christian Meyer zum Büschenfelde, Helmut Friess, Jörg Kleeff

**Affiliations:** 1 Department of Surgery, Technische Universität München, Munich, Germany; 2 Institute of Medical Statistics and Epidemiology, Technische Universität München, Munich, Germany; 3 Department of Hematology and Oncology, Technische Universität München, Munich, Germany; 4 Center of Cancer Systems Biology, Department of Medicine, Caritas St. Elizabeth's Medical Center, Tufts University School of Medicine, Boston, Massachusetts, United States of America; University of Heidelberg, Germany

## Abstract

**Background:**

Pancreatic cancer has an extremely poor prognosis and prolonged survival is achieved only by resection with macroscopic tumor clearance. There is a strong rationale for a neoadjuvant approach, since a relevant percentage of pancreatic cancer patients present with non-metastatic but locally advanced disease and microscopic incomplete resections are common. The objective of the present analysis was to systematically review studies concerning the effects of neoadjuvant therapy on tumor response, toxicity, resection, and survival percentages in pancreatic cancer.

**Methods and Findings:**

Trials were identified by searching MEDLINE, EMBASE, and the Cochrane Central Register of Controlled Trials from 1966 to December 2009 as well as through reference lists of articles and proceedings of major meetings. Retrospective and prospective studies analyzing neoadjuvant radiochemotherapy, radiotherapy, or chemotherapy of pancreatic cancer patients, followed by re-staging, and surgical exploration/resection were included. Two reviewers independently extracted data and assessed study quality. Pooled relative risks and 95% confidence intervals were calculated using random-effects models. Primary outcome measures were proportions of tumor response categories and percentages of exploration and resection. A total of 111 studies (*n* = 4,394) including 56 phase I–II trials were analyzed. A median of 31 (interquartile range [IQR] 19–46) patients per study were included. Studies were subdivided into surveys considering initially resectable tumors (group 1) and initially non-resectable (borderline resectable/unresectable) tumors (group 2). Neoadjuvant chemotherapy was given in 96.4% of the studies with the main agents gemcitabine, 5-FU (and oral analogues), mitomycin C, and platinum compounds. Neoadjuvant radiotherapy was applied in 93.7% of the studies with doses ranging from 24 to 63 Gy. Averaged complete/partial response probabilities were 3.6% (95% CI 2%–5.5%)/30.6% (95% CI 20.7%–41.4%) and 4.8% (95% CI 3.5%–6.4%)/30.2% (95% CI 24.5%–36.3%) for groups 1 and 2, respectively; whereas progressive disease fraction was estimated to 20.9% (95% CI 16.9%–25.3%) and 20.8% (95% CI 14.5%–27.8%). In group 1, resectability was estimated to 73.6% (95% CI 65.9%–80.6%) compared to 33.2% (95% CI 25.8%–41.1%) in group 2. Higher resection-associated morbidity and mortality rates were observed in group 2 versus group 1 (26.7%, 95% CI 20.7%–33.3% versus 39.1%, 95% CI 29.5%–49.1%; and 3.9%, 95% CI 2.2%–6% versus 7.1%, 95% CI 5.1%–9.5%). Combination chemotherapies resulted in higher estimated response and resection probabilities for patients with initially non-resectable tumors (“non-resectable tumor patients”) compared to monotherapy. Estimated median survival following resection was 23.3 (range 12–54) mo for group 1 and 20.5 (range 9–62) mo for group 2 patients.

**Conclusions:**

In patients with initially resectable tumors (“resectable tumor patients”), resection frequencies and survival after neoadjuvant therapy are similar to those of patients with primarily resected tumors and adjuvant therapy. Approximately one-third of initially staged non-resectable tumor patients would be expected to have resectable tumors following neoadjuvant therapy, with comparable survival as initially resectable tumor patients. Thus, patients with locally non-resectable tumors should be included in neoadjuvant protocols and subsequently re-evaluated for resection.

*Please see later in the article for the Editors' Summary*

## Abstract 2

Jörg Kleef and colleagues systematically reviewed studies on neoadjuvant therapy and tumor response, toxicity, resection, and survival percentages in pancreatic cancer and suggest that patients with locally nonresectable tumors should be included in neoadjuvant protocols.

## Editors' Summary

**Background:**

Pancreatic cancer is the fourth leading cause of cancer-related deaths worldwide. It begins when a cell in the pancreas (an organ lying behind the stomach that produces digestive enzymes and hormones such as insulin that controls blood sugar levels) acquires genetic changes that allow it to grow uncontrollably and, sometimes, to spread around the body (metastasize). Because pancreatic cancer rarely causes any symptoms early in its development, it is locally advanced in more than a third of patients and has already metastasized in another half of patients by the time it is diagnosed. Consequently, on average, people die within 5–8 months of a diagnosis of pancreatic cancer. At present, the only chance for cure is surgical removal (resection) of the tumor, part of the pancreas, and other nearby digestive organs. This procedure—the Whipple procedure—is only possible in the fifth of patients whose tumor is found when it is small enough to be resectable, and even in these patients, the cure rate associated with surgery is less than 25%, although radiotherapy or chemotherapy after surgery (adjuvant therapy) can be beneficial.

**Why Was This Study Done?:**

For patients whose tumor has metastasized, palliative chemotherapy to slow down tumor growth and to minimize pain is the only treatment option. But, for the many patients whose disease is locally advanced and unresectable at diagnosis, experts think that “neoadjuvant” therapy might be helpful. Neoadjuvant therapy—chemotherapy and/or radiotherapy given before surgery—aims to convert unresectable tumors into resectable tumors by shrinking the visible tumor and removing cancer cells that cannot be seen with the naked eye. Randomized phase III trials—studies in which groups of patients are randomly assigned to different interventions and specific outcomes measured—are the best way to determine whether an intervention has any clinical benefits, but no randomized phase III trials of neoadjuvant therapy for unresectable pancreatic cancer have been undertaken. Therefore, in this systematic review (a study that uses predefined criteria to identify all the research on a given topic) and meta-analysis (a statistical method for combining the results of several studies), the researchers analyze data from other types of studies to investigate whether neoadjuvant therapy for pancreatic cancer provides any clinical benefits.

**What Did the Researchers Do and Find?:**

In their systematic review, the researchers identified 111 studies involving 4,394 patients in which the effects of neoadjuvant chemotherapy and/or radiotherapy on tumor response, tumor resectability, and patient survival had been investigated. They subdivided the studies into two groups: group 1 studies included patients whose tumors were considered resectable on preoperative examination, and group 2 studies included patients whose tumors were borderline resectable or unresectable. In their meta-analysis, the researchers found that similar percentages of the tumors in both groups responded to neoadjuvant therapy by shrinking or regressing and that about a fifth of the tumors in each group grew larger or metastasized during neoadjuvant therapy. In the group 1 studies, three-quarters of the tumors were resectable after neoadjuvant therapy (a decrease in the proportion of tumors that could be treated surgically) whereas in the group 2 studies, a third of the tumors were resectable after neoadjuvant therapy (an increase in the proportion of tumors that could be treated surgically). After resection, the average survival time for group 1 patients was 23.3 months, a similar survival time to that seen in patients treated with surgery and adjuvant therapy. The average survival time for group 2 patients after resection was 20.5 months.

**What Do These Findings Mean?:**

The finding that the average survival time after neoadjuvant therapy and surgery in patients whose tumor was judged resectable before neoadjuvant therapy was similar to that of patients treated with chemotherapy and/or radiotherapy after surgery suggests that for patients with resectable tumors, neoadjuvant therapy will not provide any clinical benefit. By contrast, the finding that a third of patients initially judged unresectable were able to undergo resection after neoadjuvant therapy and then had a similar survival rate to patients judged resectable before neoadjuvant treatment strongly suggests that patients presenting with locally advanced/unresectable tumors should be offered neoadjuvant therapy and then re-evaluated for resection. Randomized trials are now needed to confirm this finding and to determine the optimum neoadjuvant therapy for this group of patients.

**Additional Information:**

Please access these Web sites via the online version of this summary at http://dx.doi.org/10.1371/journal.pmed.1000267.

- The US National Cancer Institute provides information for patients and health professionals about all aspects of pancreatic cancer (in English and Spanish), including a booklet for patients
- The American Cancer Society also provides detailed information about pancreatic cancer
- The UK National Health Service and Cancer Research UK include information for patients on pancreatic cancer on their Web sites
- MedlinePlus provides links to further resources on pancreatic cancer (in English and Spanish)
- Pancreatica.org, PancreaticDuct.org, and the Pancreatic Cancer Action Network give more information to pancreatic cancer patients, their families, and caregivers

## Introduction

Pancreatic ductal adenocarcinoma (PDAC) is the fourth leading cause of cancer-related mortality [1] and is associated with an extremely poor prognosis, reflected by a median survival of 5–8 mo and a 5-y survival probability of less than 5% when all stages are combined [1]–[3]. At present, the only chance for cure and prolonged survival is surgical resection with macroscopic tumor clearance. However, only approximately 10%–20% [1],[4] of patients are considered candidates for curative resection. The majority of patients (50%–60%) present with metastatic disease, and thus palliative chemotherapy remains the only option for almost all of these patients [5].

In a substantial number of patients (approximately 30%–40%) the disease is considered “locally advanced” at the time of diagnosis. This group of patients has been intensively discussed during the last years and neoadjuvant therapies have been proposed to achieve better local tumor control or tumor down-staging with a subsequent potentially resectable tumor [6]. Neoadjuvant therapy in this context is defined as any preoperative therapy aiming to convert unresectable to resectable tumors and/or to increase microscopic complete tumor resection rates. Unfortunately, however, no data regarding the role of neoadjuvant therapy for pancreatic cancer from randomized phase III trials are available. In addition, a thorough analysis of this group of patients has been hampered by the lack of an accepted and widely used definition of resectability and unresectability. For example, while current guidelines generally consider encasement/involvement of the superior mesenteric artery/celiac trunk as signs of unresectability [7],[8], portal vein/superior mesenteric vein involvement has been more critically discussed [9] and categories such as “borderline resectable” have been introduced [8]. Furthermore, all criteria depend heavily on the experience and technical expertise of the involved radiologists, gastroenterologists, and surgeons.

Even following potential curative resection more than 80% of the patients ultimately die of the disease due to local recurrence and/or distant metastasis. The high rate of local recurrence [10] is predetermined by the microscopic frequently incomplete resections [11]–[13] as a result of the anatomical location of the tumor and the growth pattern of pancreatic cancer cells. Adjuvant therapy [14]–[16] has been established as the standard of care following resection for pancreatic adenocarcinoma. Here, solid data from randomized controlled trials [17]–[22] suggest that adjuvant chemotherapy (gemcitabine or 5-FU) is the standard treatment option. In contrast, there is still discussion regarding the role of adjuvant chemoradiation [20],[23] specifically in the subgroup of patients with positive resection margins [15].

The relatively high percentage of PDAC patients presenting with non-metastatic but “locally advanced” disease as well as the large number of microscopic incomplete resections [11],[13] should provide a strong rationale for a neoadjuvant approach. Although neoadjuvant therapy for pancreatic cancer has been proposed for more than two decades [24],[25], and although there is strong evidence of its benefit for other tumor entities, up to now there is no compelling evidence for a clinical benefit of neoadjuvant therapy in pancreatic cancer. Here, we systematically reviewed and performed a meta-analysis of the available data regarding neoadjuvant chemo- and/or radiotherapy with special emphasis on tumor response/progression rates, toxicities, and clinical benefit, i.e. resection probabilities and survival estimates.

## Methods

Although no randomized phase III trials could be considered within this review, general recommendations from QUOROM [26] and the PRISMA revision [27] with regard to processing and reporting of results were taken into account (Text S1).

### Trial Criteria

This systematic review and meta-analysis incorporated retrospective and prospective studies of patients with pancreatic and periampullary cancer with the following design: neoadjuvant radiochemotherapy, radiotherapy, or chemotherapy, followed by re-staging, and surgical exploration/resection in selected patients. Phase I–II clinical trials, cohort studies, and case series were included. Case reports were excluded as were reports of identical patient cohorts (if clearly identifiable).

### Search Strategy

Trials were identified by searching MEDLINE, EMBASE, and the Cochrane Central Register of Controlled Trials from 1966 to December 2009 (Text S2). The search strategy included the following search keys: (“pancreas” or “pancreatic”) and (“cancer” or “carcinoma”) and (“neoadjuvant” or “preoperative”) and (“radiation” or “chemoradiation” or “chemotherapy”), without language restriction. The results were then hand-searched for eligible studies. Furthermore we searched the proceedings of the Gastrointestinal Cancers Symposium and ASCO Annual Meeting from 2004 to December 2009. In addition, reference lists of the selected trials were screened for any other relevant study. Prospective and ongoing trials were identified by searching the following prospective trials registers and databases (last search December 1, 2009): ISRCTN Register, Action Medical Research, Leukaemia Research Fund, Medical Research Council (UK), National Health Service Research and Development Health Technology Assessment Programme (HTA), National Institutes of Health (www.ClinicalTrials.gov), The Wellcome Trust and the UK Clinical Trials Gateway, and The WHO International Clinical Trials Registry Platform (www.who.int/ictrp) including the Australian New Zealand Clinical Trials Registry (ANZCTR), Chinese Clinical Trial Register (ChiCTR), Clinical Trials Registry–India (CTRI), German Clinical Trials Register (DRKS), Iranian Registry of Clinical Trials (IRCT), Sri Lanka Clinical Trials Registry (SLCTR), and The Netherlands National Trial Register (NTR). The search strategy for these trials included the following search keys: ((“pancreas” or “pancreatic”) and (“cancer” or “carcinoma”) and (“neoadjuvant” or “preoperative”)) or ((“pancreas” or “pancreatic”) and (“cancer” or “carcinoma”) and (“non-metastatic” or “nonmetastatic”) and (“unresectable” or “non-resectable” or “locally advanced”)), without language restriction.

### Selection of Trials and Data Collection

Two reviewers (SG, JK) independently assessed the eligibility of abstracts identified by the search. The full-text article of any trial that appeared to meet the inclusion criteria was retrieved for closer examination. Disagreements were resolved by consensus. The same reviewers extracted the data independently using standardized data collection forms. Data retrieved from the reports include publication details (year of publication, study center), methodological components, and trial characteristics, such as sample size, interventions (radiochemotherapy, radiotherapy, or chemotherapy), and outcome measures (Text S2). Included studies were subdivided into three groups: those studies analyzing patients with pancreatic and periampullary cancer who were judged resectable on preoperative staging (group 1), those studies analyzing patients whose tumors were judged borderline resectable or unresectable (subsequently termed non-resectable; group 2), and those studies that included all patients with localized non-metastatic disease (resectable and non-resectable). Studies were analyzed with respect to the utilized resectability criteria and grouped according to the current National Comprehensive Cancer Network (NCCN) criteria for resectability [28] if applicable. In cases where resectability criteria were not or not clearly stated, tumors were grouped according to the stated resectability category.

### Outcome Measures

The primary outcome measures were proportions of tumor response categories (CR, PR, SD, PD) as well as percentages of exploration and resection. Secondary outcome measures included toxicity, morbidity, mortality, and survival. The authors aimed to unify definitions of tumor response across studies in accordance with the RECIST criteria [29]: Complete response: disappearance of all target lesions (radiographic) or no vital tumor cells (histopathological); partial response: 30% decrease of the target lesion (radiographic) or marked signs of tumor regression (histopathologic); progressive disease: 20% increase of the target lesion (radiographic), or distant metastases (radiographic or histopathologic); stable disease: no change or small changes that did not meet the above criteria. In case of discrepant radiological and histopathological response, the histopathological response was taken for calculation.

### Quality Assessment

To assess the overall strength/quality of evidence for the various outcome parameters, a quality assessment was carried out in the style of the GRADEprofiler (GRADEpro. [Computer program]. Version 3.2 for Windows. Jan Brozek, Andrew Oxman, Holger Schünemann, 2008). Study design, study limitation, risk of bias, study inconsistency, indirectness, and imprecision were rated according to the GRADEprofiler. Study quality was classified as high, moderate, low, or very low. Outcome parameters were classified as critical, important but not critical, or of limited importance.

### Statistical Analysis

The statistical software package *R* version 2.7.1 (R Foundation for Statistical Computing, Vienna, Austria) with function *metaprop* (R package: meta, *Schwarzer* 2008) was used for the statistical analyses. Pooled estimates of proportions with corresponding 95% confidence intervals were calculated on the base of the Freeman-Tukey double arcsine transformation [30],[31] within a random effect model framework. Heterogeneity of combined study results was assessed by inconsistency statistic (I^2^) and its connected chi-square test for heterogeneity, and I^2^ and the corresponding 95% confidence intervals were calculated. No formal test was conducted for purpose of subgroup comparisons and results were solely displayed in a comparative descriptive manner. References from literature [32] as well as especially conducted preliminary simulation studies suggest that unbiased pooled estimates of median survival times cannot be achieved by simple weighted averaging of medians. A more appropriate approach is achieved by averaging parameter estimates of a presumed density function of survival and recalculating the estimate of median from the pooled distribution parameter. A reasonable distribution of survival times which implies a time constant hazard rate corresponding to the sole distribution parameter λ is given by the *exponential distribution*. Following this assumption, a weighted estimate of population median (*m_p_*) survival is derived by:
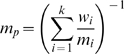
where *m_i_* denotes the median survival within a study population *i* (with *i* from 1 to *k*) and *w_i_* refers a study specific weight function, and Σ*wi* = 1. Since no sufficient information on patients at risk for median survival times was available from the considered studies, number of study participants (divided by the total number of evaluable patients) was used as weights. Confidence intervals for median estimates were not calculable and therefore the range of medians was provided instead of confidence limits. In order to analyze potential publication bias, funnel plots were created for the outcome parameters using a Web-based software tool (*Eastern Region Public Health Observatory* (erpho); tools.erpho.org.uk/binomial.aspx). Graphs were plotted using GraphPad Prism 5 for Windows (GraphPad, San Diego, CA).

### Analysis of Heterogeneity

The general linear modeling framework to extract sources of variance (heterogeneity) from the study data was used. For this purpose, variables potentially explaining clinical incomparability and design incomparability, respectively, were considered in the meta-regression analysis following the terminology of Thompson [33]. These variables were resectability (yes, no, both, or not defined), resectability criteria (NCCN criteria [28], clearly defined criteria, not clearly defined, or no stated criteria), mean age of patients (5-y intervals), mean year of study interval (decades), chemotherapy (no, monotherapy, combination therapy), institution and study design (phase I, phase I/II, phase II, cohort study, case series, retrospective, prospective), and evaluation criteria (RECIST criteria [29], clearly defined criteria, not clearly defined, or no stated criteria). For purpose of a reliable statistical analysis, arcsine transformation was applied first to the main outcome parameters (proportions, e.g. fraction of resected and explored patients, respectively). The number of patients for each specific outcome parameter was considered as residual weight within the regression model and the fraction of explained variance was gathered for each model component. The main outcome parameters were (transformed) proportions; therefore percentages of explained variability have to be interpreted in a relative (comparative) manner rather than in an absolute one.

## Results

**Figure 1:**
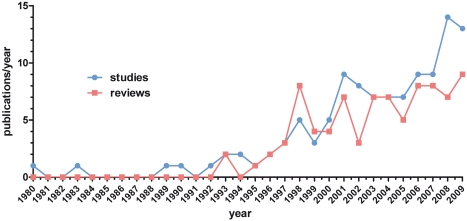
Number of identified original studies (*n* = 111) and reviews (*n* = 85) per year (1980–2009).

From 515 initially retrieved studies, 111 studies published since 1980 were identified and included in this systematic review and meta-analysis (Figure S1, Table S1). Four studies overlapped with four of the 111 studies, and were therefore excluded. In the same time period, 85 reviews regarding neoadjuvant therapy in pancreatic cancer were published. The number of published original and review articles increased steadily within the last 15 years (Figure 1).

The 111 reviewed trials included 4,394 patients. Seventeen centers published more than 1 study, altogether accounting for 73 of the 111 studies (Table S1). Eight of the 17 centers published 2 studies. The University of Texas M.D. Anderson Cancer Center (Houston, TX) and the Fox Chase Cancer Center (Philadelphia, PA) published 12 and 11 studies, respectively (Table S1). Other centers such as University of Osaka (Japan), the University of Marseille (France), and the Duke University (Durham, NC) published 8, 6, and 5 studies, respectively. The potentially overlapping patient populations were difficult to calculate since the information of which patients were included in which analysis could not always be retrieved. Using the study periods, study protocols, and a conservative estimate, there was a maximum of 17% overlapping patient populations.

Of the 111 included studies, 78 studies were prospective and 33 retrospective. There were 15 phase I, 13 phase I/II, and 28 phase II studies, as well as 14 cohort studies and 41 case series. No phase III trials have been published so far. A systematic search of clinical trial databases for pancreatic cancer trials identified 17 neoadjuvant trials (all phase I–II trials) and 23 trials for non-resectable but non-metastatic pancreatic cancer, i.e. potentially neoadjuvant trials (Table S2).

The 111 analyzed studies reported on PDAC that generally included pancreatic head, corpus, and tail tumors without separate analysis regarding tumor localization. The studies included a median (IQR) of 31 (19–46) patients (Table 1). Ten of the 111 studies included in addition to pancreatic cancer a few patients with other periampullary tumors (i.e. ampullary, distal bile duct, and duodenal cancer), without separate analysis of the different entities. In 84 studies (76%), it was explicitly stated that histological or cytological tumor diagnosis was obtained before therapy. The age of the included patients varied, as did its reporting. The median of reported age of the patients in the 94 assessable studies was 62.5 y and was similar in the analyzed groups (group 1: 62 y, group 2: 62 y).

**Table 1:** Summary of included studies in the different defined groups.

Group	Total Number of Studies (%)	Patients per Study Median (IQR)
All patients	111	31 (19–46)
Group 1 (tumor resectable before treatment)	35 (31.5%)	32 (20–50)
Group 2 (tumor non-resectable before treatment)	57 (51.4%)	27 (18–38)
Group 3 (both or not defined)	19 (17.1%)	38 (24–82)

### Chemotherapy

**Figure 2:**
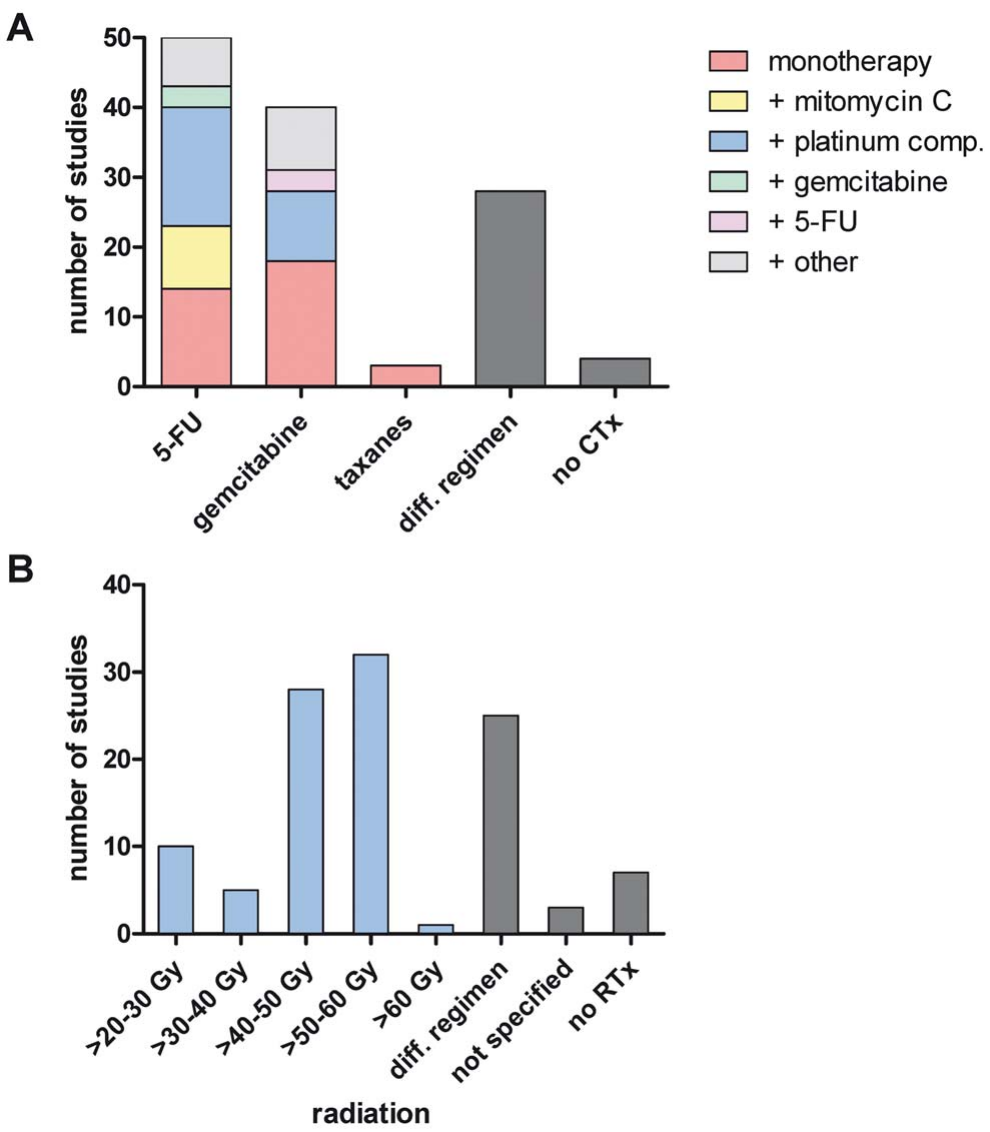
Depiction of utilized chemotherapy and radiotherapy. (A) Schematic overview of the used chemotherapeutic agents. diff. regimen, studies comparing/using different drug regimen (*n* = 28); no CTx, no chemotherapy applied (only radiotherapy). (B) Schematic overview of the applied radiation doses. Studies are summarized within a range of applied doses. Data included are per protocol, not all patients received the stated dose. diff. regimen, different radiation doses applied; not specified, radiation applied, dose not specified; no RTx, no radiotherapy applied (only chemotherapy).

Chemotherapy was applied as neoadjuvant treatment in 107 of the 111 studies (96.4%). Different combinations of chemotherapies/agents and dosages were tested, as 56 of the studies were phase I–II trials. The main agents were gemcitabine, 5-FU (and oral analogues), mitomycin C, and platinum compounds (Figure 2A). In the trials that used only one regimen (*n* = 79), 43 (54.4%) were performed using 5-FU or its oral analogues. 5-FU monotherapy was given in 14 (17.7%) of the studies. Thirty-six (45.6%) of the studies used a gemcitabine-based regimen, and of those, 18 (22.8%) studies applied gemcitabine monotherapy. 5-FU and gemcitabine combinations were used in 3 studies. Several studies compared different schemes or agents. Five studies were performed comparing gemcitabine with 5-FU or capecitabine, two studies comparing gemcitabine with cisplatin, two gemcitabine with 5-FU/cisplatin, and another three gemcitabine with 5-FU/mitomycin C. A further 16 studies included different agents and combinations (some for only few patients) (Table S1). Twelve trials included taxanes (docetaxel/paclitaxel) in different combinations or as monotherapy (*n* = 3). Five of the 107 studies included antibodies or tyrosine kinase inhibitors (bevacizumab, cetuximab, erlotinib) in the chemotherapeutic regimen. There were 44 studies using single agents (alone or in comparison) and 48 studies using combination therapies. In 15 studies both single agents and combination therapies were utilized.

### Radiotherapy

In 104 of the 111 studies (93.7%) patients received neoadjuvant radiotherapy. In three studies the exact radiation dose was not given. Doses applied ranged from 24 Gy to 63 Gy (Figure 2B). In 52 of the 104 studies that included radiotherapy the patients received doses between 45 and 50.4 Gy. In 14 studies different doses and radiation schedules were compared. Most patients received 1.8 Gy/fraction (50/104 studies), 2 Gy/fraction (15/104), or 3 Gy/fraction (10/104). In 13 studies intraoperative radiation (IORT) was applied with doses between 10 and 30 Gy. Since in most of those studies only few patients received IORT, this aspect was not further analyzed.

### Toxicity

Data regarding treatment-related toxicity were available for 63 of 111 studies. For subsequent analysis, only severe (grade 3/4) toxicity (National Cancer Institute Common Toxicity Criteria; ctep.cancer.gov) was taken into account. Grade 3/4 toxicity for neoadjuvant therapy was estimated at 29.4% (CI 23.1%–36.1%) for all patients and was comparable for initially resectable (26.3%, CI 15.8%–38.3%) and patients with non- resectable tumors (“non-resectable tumor patients”) (31.1%, CI 22%–40.9%) (Table 2). Recent randomized controlled trials for adjuvant therapy report grade 3/4 toxicity rates for chemotherapy of 8.4%–22% (only neutropenia [21]) and 14.7% (all toxicity [20]). The reported grade 3/4 toxicity rates for radiochemotherapy were 9%–58% (only hematological toxicity [34]) and 22.2%–79% (all toxicity [20]).

**Table 2:** Estimates of grade 3/4 toxicity of neoadjuvant treatment including the 95% confidence interval from the random effect model and number of assessable studies for each group (*n*).

Group	Grade 3/4 Toxicity
All patients	**29.4%** [23.1%–36.1%]I^2^ = 91.3% [89.6%–92.7%](*n* = 63)
Tumor resectable before treatment (group 1)	**26.3%** [15.8%–38.3%]I^2^ = 92.8% [90.3%–94.6%](*n* = 22)
Tumor non-resectable before treatment (group 2)	**31.1%** [22.0%–40.9%]I^2^ = 91.6% [89.3%–93.5%](*n* = 33)

### Tumor Response

**Figure 3:**
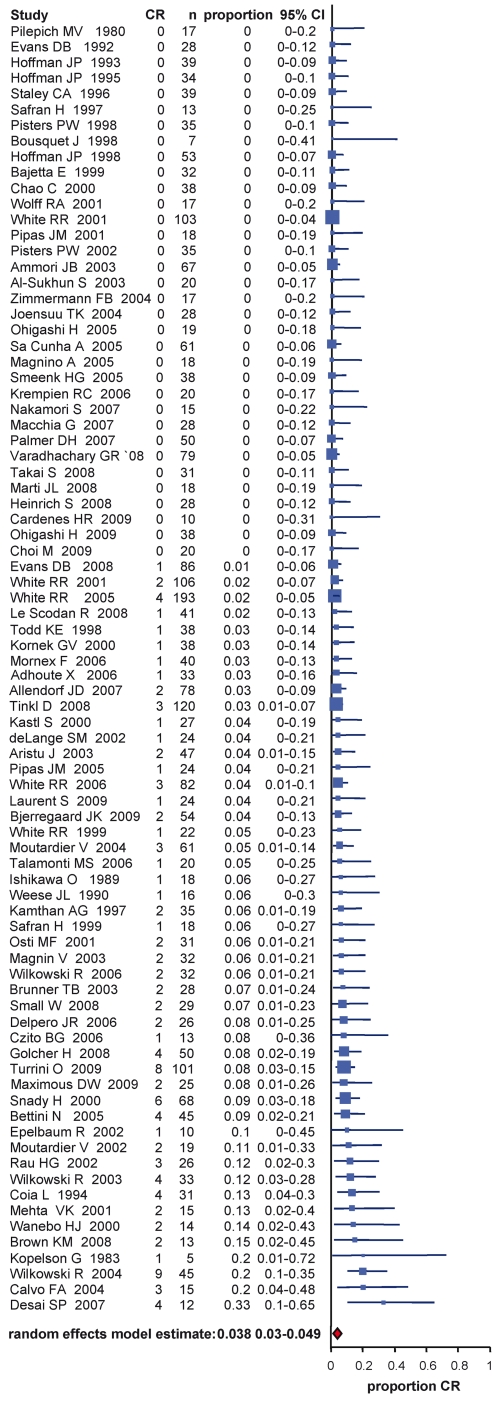
Estimates of complete response percentages in patients following neoadjuvant therapy and re-staging including the 95% confidence interval from the random effect model and number of patients for each study (*n*).

**Figure 4:**
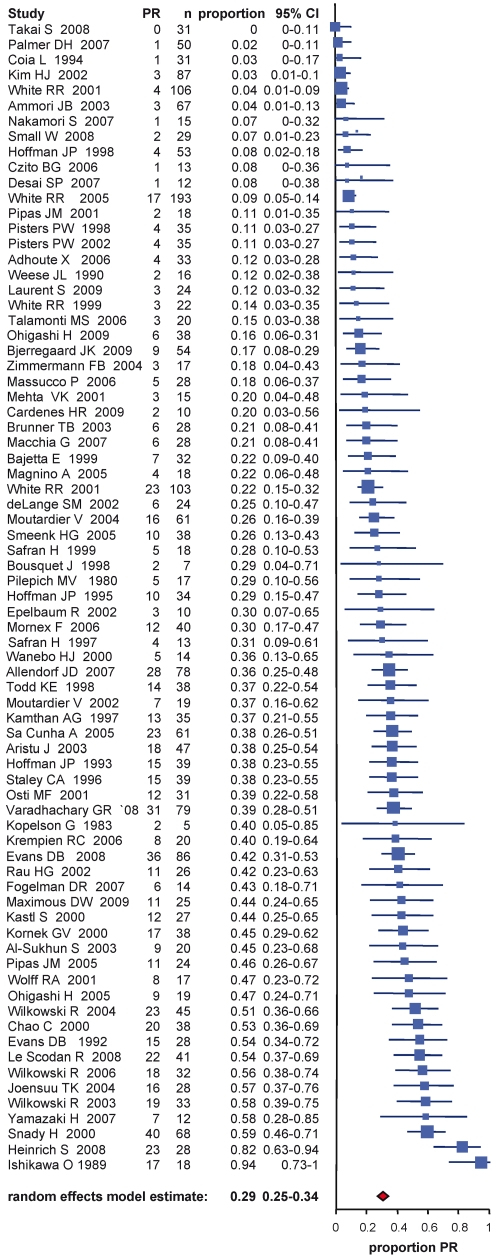
Estimates of partial response percentages in patients following neoadjuvant therapy and re-staging including the 95% confidence interval from the random effect model and number of patients for each study (*n*).

Tumor response frequency for neoadjuvant chemo- and/or radiation therapy was evaluated in the different studies according to either radiographic or clinical response evaluation before exploration or histopathological response after resection. Six studies (5.4%) explicitly stated that the RECIST criteria [29] were utilized. In 44 studies (39.6%) the criteria to assess tumor response were clearly stated, whereas in 61 studies (55%) criteria were either not clearly defined or not stated. For the whole study population the estimated fraction of patients with complete response was 3.9% (CI 3%–4.9%) (Figure 3) and with partial response 29.1% (CI 24.5%–34%) (Figure 4). Stable disease was averaged to 43.9% (CI 37.9%–50%) in all patients and tumor progression under therapy occurred by estimation in 20.8% (CI 17.3%–24.6%) of the patients. Interestingly the pooled percentages did not vary much in the two groups of initially deemed resectable and non-resectable tumor patients (Table 3). Thus, complete/partial responses were 3.6%/30.6% and 4.8%/30.2% for groups 1 and 2, respectively; whereas progressive disease was estimated to 20.9% (CI 16.9%–25.3%) and 20.8% (CI 14.5%–27.8%) of primarily staged resectable and non-resectable tumor patients. Comparing tumor response frequencies for patients treated with mono chemotherapy (*n* = 44) versus combination chemotherapy (*n* = 48) revealed complete and partial responses of 2.2% (CI 1.3%–3.3%) and 25.8% (CI 20.2%–31.8%) versus 5.3% (CI 3.8%–7%) and 34.7% (CI 28.9%–40.9%) (Table 4).

**Table 3:** Estimates of exploration and resection percentages after neoadjuvant treatment and restaging, and estimates of patients with complete response/partial response, stable disease, and progressive disease including the 95% confidence interval from the random effect model and number of assessable studies for each group (*n*).

Group	Complete Response	Partial Response	Stable Disease	Progressive Disease	Explored/All	Resected/All	R0/Resected	Resected/Explored
All patients	3.9%[3.0%–4.9%]I^2^ = 44.7%[28.1%–57.5%](*n* = 82)	29.1%[24.5%–34.0%]I^2^ = 86.9%[84.2%–89.1%](*n* = 75)	43.9%[37.9%–50.0%]I^2^ = 87.7%[85%–89.9%](*n* = 63)	20.8%[17.3%–24.6%]I^2^ = 81.4%[77.3%–84.8%](*n* = 78)	69.5%[62.1%–76.4%]I^2^ = 95.5%[94.9%–96%](*n* = 88)	50.7%[44.0%–57.4%]I^2^ = 95.2%[94.6%–95.7%](*n* = 111)	79.6%[74.8%–83.9%]I^2^ = 81.3%[77.4%–84.6%](*n* = 86)	77.9%[72.4%–82.9%]I^2^ = 89%[87%–90.6%](*n* = 88)
Tumor resectable before treatment (group 1)	3.6%[2.0%–5.5%]I^2^ = 53.9%[29.3%–70%](*n* = 28)	30.6%[20.7%–41.4%]I^2^ = 90.3%[86.7%–92.9%](*n* = 23)	42.1%[30.5%–54.1%]I^2^ = 91.4%[88.4%–93.6%](*n* = 23)	20.9%[16.9%–25.3%]I^2^ = 66.9%[51.2%–77.5%](*n* = 29)	88.1%[82.9%–92.4%]I^2^ = 86.2%[81.5%–89.7%](*n* = 32)	73.6%[65.9%–80.6%]I^2^ = 90.1%[87.3%–92.3%](*n* = 35)	82.1%[73.1%–89.6%]I^2^ = 89.3%[85.5%–92%](*n* = 26)	85.7%[78.9%–91.2%]I^2^ = 88.6%[85%–91.4%](*n* = 32)
Tumor non-resectable before treatment (group 2)	4.8%[3.5%–6.4%]I^2^ = 33.9%[3.4%–54.8%](*n* = 42)	30.2%[24.5%–36.3%]I^2^ = 81.8%[75.9%–86.2%](*n* = 40)	41.6%[34.6%–48.7%]I^2^ = 75%[64.2%–82.6%](*n* = 29)	20.8%[14.5%–27.8%]I^2^ = 85.4%[80.7%–88.9%](*n* = 36)	46.9%[36.9%–57.1%]I^2^ = 93.7%[92.2%–94.8%](*n* = 41)	33.2%[25.8%–41.1%]I^2^ = 92.5%[91%–93.7%](*n* = 57)	79.2%[72.4%–85.2%]I^2^ = 70.2%[59.7%–78%](*n* = 45)	69.9%[61.2%–77.9%]I^2^ = 79.9%[73.3%–84.9%](*n* = 41)

**Table 4:** Estimates of percentage of responses and resections in patients receiving mono chemotherapy versus combination chemotherapy groups including the 95% confidence interval from the random effect model and number of assessable studies for each group (*n*).

Group	Mono Chemotherapy	Combination Chemotherapy
Complete response[95% CI](number of studies assessable)	2.2% [1.3%–3.3%]I^2^ = 20.8% [0%–49.7%](*n* = 30)	5.3% [3.8%–7.0%]I^2^ = 48.3% [25.5%–64.1%](*n* = 41)
Partial response[95% CI](number of studies assessable)	25.8% [20.2%–31.8%]I^2^ = 78.8% [70.3%–84.9%](*n* = 30)	34.7% [28.9%–40.9%]I^2^ = 79.5% [72.1%–85%](*n* = 35)
Resection rate (group 1)[95% CI](number of studies assessable)	80.8% [66.1%–92.1%]I^2^ = 93.9% [91.2%–95.7%](*n* = 13)	66.2% [57.9%–74.0%]I^2^ = 77.1% [62.6%–86%](*n* = 19)
Resection rate (group 2)[95% CI](number of studies assessable)	27.3% [18.1%–37.5%]I^2^ = 87.7% [82.7%–91.3%](*n* = 22)	33.0% [25.2%–41.3%]I^2^ = 87.3% [82.9%–90.6%](*n* = 29)

### Exploration and Resection

Operations performed included explorative laparotomies, palliative bypass procedures, and curative resections, e.g. partial pancreatico-duodenectomies, distal pancreatectomies, and total pancreatectomies. Studies were analyzed for patients explored and resected after restaging. All 111 studies included data for resection.

**Figure 5:**
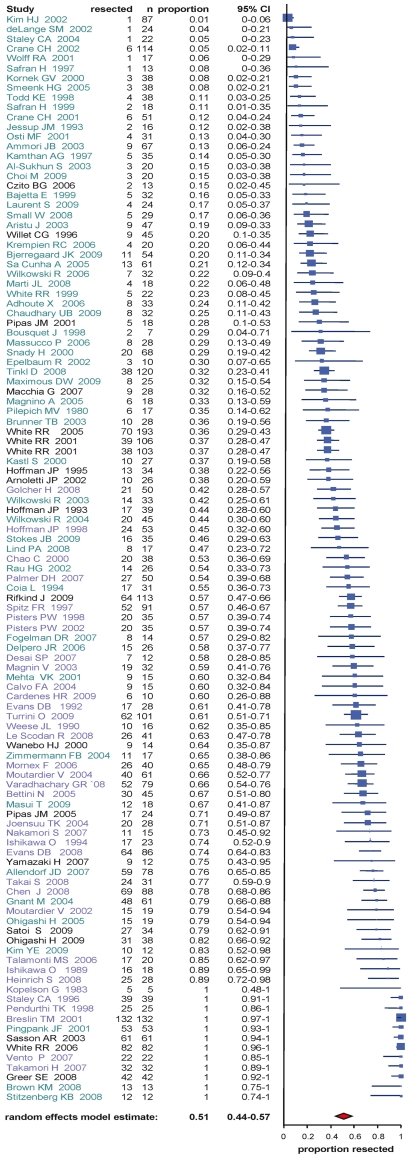
Estimates of resection percentages in patients following neoadjuvant therapy and re-staging including the 95% confidence interval from the random effect model and number of patients for each study (*n*). Studies analyzing initially resectable tumor patients are depicted in blue, initially non-resectable tumor patients in green, and those including both (or not defined) in black.

**Figure 6:**
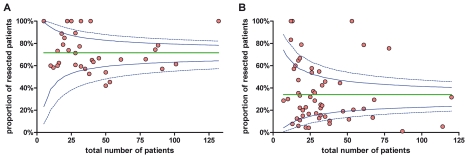
Funnel plots for the resection rate for studies analyzing initially resectable (A) and non-resectable (B) tumor patients. Green line, average proportion; dashed blue line, upper and lower 3 SD limits; solid blue line, upper and lower 2 SD limits.

Seven studies (6.3%) explicitly used the NCCN guidelines of resectability for non-metastatic pancreatic cancer [28]. Forty-five studies (40.5%) clearly defined the resectability criteria assessing most often the vascular involvement or classified the resectability according to the maximal tumor dimension. In 59 studies (53.2%), resectability criteria were not clearly stated (e.g. judged by single surgeons or an interdisciplinary team) or not stated at all. In group 1 including the patients who were staged to be resectable before neoadjuvant treatment resectability estimated to 73.6% (CI 65.9%–80.6%) (Figure 5, Table 3), whereas in group 2 including the patients who were staged non-resectable before treatment the averaged probability for resectability was 33.2% (CI 25.8%–41.1%) (Figure 5, Table 3). As shown in Table 3, in the assessable studies the percentage of exploration for the entire group was 69.5% (CI 62.1%–76.4%) and 77.9% (CI 72.4%–82.9%) of these patients were resected. Of the patients deemed resectable before treatment, 88.1% (CI 82.9%–92.4%) were explored after restaging, and of those 85.7% (CI 78.9%–91.2%) could be resected. In group 2, 46.9% (CI 36.9%–57.1%) of the patients were explored. Of them, 69.9% (CI 61.2%–77.9%) could be resected successfully (Table 3). Interestingly the estimated fraction of R0 resections were comparable between patients in group 1 (82.1%; CI 73.1%–89.6%) and patients in group 2 (79.2%; 72.4%–85.2%) (Table 3). To analyze potential publication bias, funnel plots were created (Figure 6) that demonstrated heterogeneity (see below) but no considerable imbalance (no reasonable evidence for publication bias) neither for the group of patients with initially resectable tumors (“resectable tumor patients”) nor for the non-resectable tumor patients. There were three considerable outliers in the non-resectable group (Figure 6B). Omission of these trials in another supportive meta-analysis regarding resection rates demonstrated an estimated resection probability of 30% (CI 24%–36%), which was similar to the estimated proportion of 33% (CI 26%–41%) for the entire group of non-resectable tumor patients.

Analyzing resection frequencies for patients treated with mono chemotherapy versus combination chemotherapy revealed that in the group of initially resectable tumor patients, the averaged fraction of resections for patients receiving monotherapy was 80.8% (CI 66.1%–92.1%) and for combination chemotherapy 66.2% (CI 57.9%–74%). In contrast, in patients with locally advanced/unresectable tumors, resections were more frequent in the group of patients who received combination chemotherapy with 33% (CI 25.2%–41.3%) in comparison to monotherapy with 27.3% (CI 18.1%–37.5%) (Table 4).

### Morbidity and Mortality

Data regarding morbidity and mortality following neoadjuvant treatment and pancreatic resection were presented in 50 and 85 of 111 studies, respectively. Perioperative morbidity was estimated at 34.2% (CI 28.3%–40.4%) for all patients (Table 5), which is within the range of reported morbidity data of 30%–55% for major pancreatic (head) resections [35]. In-hospital mortality after neoadjuvant treatment and tumor resection was estimated at 5.3% (CI 4.1%–6.8%) for all patients (Table 5), which is at the upper limit of the 2%–5% mortality rates that have been reported in large series and surveys for major pancreatic resections at high volume centers [35]–[37]. Interestingly, morbidity and mortality rates were estimated higher in the group of initially non-resectable versus resectable tumor patients (morbidity: 39.1% versus 26.7%, mortality: 7.1% versus 3.9%) (Table 5).

**Table 5:** Estimates of morbidity and mortality in patients undergoing pancreatic resection following neoadjuvant therapy including the 95% confidence interval from the random effect model and number of assessable studies for each group (*n*).

Group	Morbidity	Mortality
All patients	34.2%[28.3%–40.4%]I^2^ = 75.8%[68.2%–81.5%](*n* = 50)	5.3%[4.1%–6.8%]I^2^ = 29.2%[7%–46.1%](*n* = 85)
Tumor resectable before treatment (group 1)	26.7%[20.7%–33.3%]I^2^ = 67.2%[48.8%–79%](*n* = 22)	3.9%[2.2%–6.0%]I^2^ = 51.9%[26.9%–68.3%](*n* = 30)
Tumor non-resectable before treatment (group 2)	39.1%[29.5%–49.1%]I^2^ = 67.5%[49.8%–78.9%](*n* = 23)	7.1%[5.1%–9.5%]I^2^ = 0%[0%–23.4%](*n* = 43)

### Survival Analysis

**Figure 7:**
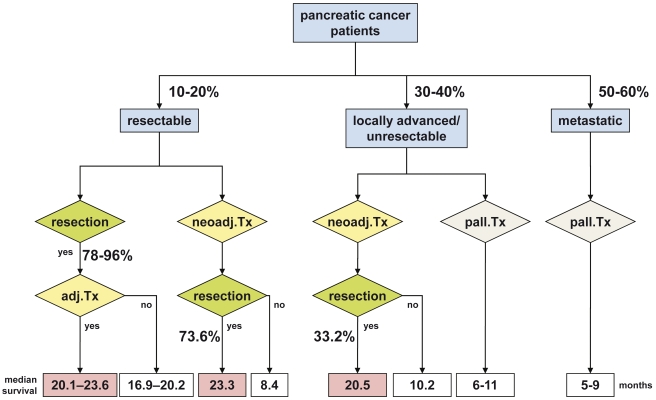
Summary overview of survival and resection percentages of different groups of patients with pancreatic cancer. Note that survival estimates derive from this systematic review and referenced studies.

Estimates of population median survival times were calculated as described and are provided with ranges from evaluable studies. Survival times for the individual studies were calculated from the time of diagnosis/start of neoadjuvant therapy in 47 trials and from surgery/resection in 4 trials. In 60 studies no detailed information regarding survival or survival calculations were provided. The longest median survival (23.3 mo, range 12–54 months) was estimated for the group of initially staged resectable tumor patients who were resected after neoadjuvant treatment (Table 6, Figure 7). The initially non-resectable staged patients reached an estimated median survival of 20.5 (range 9–62) mo following resection. The estimated median survival for the entire group of resected patients was 22.4 (range 9–62) mo. As expected, the median survival of the entire group of patients who did not undergo resection was shorter with 9.5 (range 6–21) mo. The patients who were initially classified as resectable and did not undergo resection after pretreatment survived an estimated median of 8.4 (range 6–14) mo, compared to 10.2 (range 6–21) mo of patients initially diagnosed as unresectable who did not undergo resection. Estimated 1- and 2-y survival probabilities for resected patients in group 1 were 77.9% and 47.4% and for group 2 79.8% and 50.1% (Table 6).

**Table 6:** Estimates of median survival times (*m_p_*) in months and survival probabilities.

Group	Estimated Median Survival (*m_p_*)		Estimated Survival Probability (Resected)	
	Resected (Range)	Not Resected (Range)	1 Year (Range)	2 Year (Range)
All patients	22.4(9–62)(*n* = 70)	9.5(6–21)(*n* = 51)	78.9% (0%–100%)I^2^ = 48.1% [28.7%–62.3%](*n* = 54)	49.2% (0%–82%)I^2^ = 85.2% [80.5%–88.7%](*n* = 37)
Tumor resectable before treatment (group 1)	23.3(12–54)(*n* = 27)	8.4(6–14)(*n* = 19)	77.9% (48%–100%)I^2^ = 70.7% [52.6%–81.8%](*n* = 18)	47.4% (25%–70%)I^2^ = 69.1% [42.2%–83.4%](*n* = 11)
Tumor non-resectable before treatment (group 2)	20.5(9–62)(*n* = 29)	10.2(6–21)(*n* = 25)	79.8% (0%–100%)I^2^ = 92.1% [89.8%–93.9%](*n* = 29)	50.1% (0%–82%)I^2^ = 88.6% [84%–91.9%](*n* = 21)

*n*, number of assessable studies for each group.

### Analysis of Heterogeneity and Quality Assessment

The results of meta-regression, particularly the amount of explained heterogeneity (variance components), are summarized in Table 7. For the purpose of sensitivity analysis, particularly to investigate sensibility of results with regard to model considerations (prospective under- and/or over-fitting), both univariable and multivariable models (simultaneously including all potential explanatory factors) were employed. In total, 17 institutions could be identified which contributed more than one study to the total number of trials. There were two institutions with more than 10 trials considered in our systematic review and meta-analysis. The results of the multivariable meta-regression analysis revealed that the amount of heterogeneity could be explained from about 13% to 35% by differences between institutions (considered as random effect variable). The highest impact of centers was observed regarding toxicity (34.8%) despite simultaneous consideration of chemotherapy, which corresponds to the next highest component of total variability at least in the multivariable analysis. Study design showed some impact on response evaluation (explained variability: up to 11%) and morbidity (up to 9%). Mean age of patients and study period are considerable explanatory variables for heterogeneity in morbidity and in-hospital mortality with an estimated amount of explained heterogeneity of about 10%. Heterogeneity of resection and exploration rates could mainly be deduced to resectability as well to differences between institutions and there was no sufficient explanation supported by the other potentially influencing variables. In general, the results of univariable and multivariable heterogeneity analysis were quite comparable. However, some range in attributable source of outcome variability was apparent for resectability (concerning resection and exploration rates), institution (concerning response rates), and chemotherapy (concerning complete response rates and toxicity) (Table 7). Quality assessment according to the GRADEprofiler regarding toxicity analysis, response evaluation, resection and exploration rates, morbidity, mortality, and survival analysis is presented in Table 8.

**Table 7:** Multivariable meta-regression analysis for different variables as indicated and described in the Methods section.

Potential Explanation Factor Variable	Resection Rate	Exploration Rate	CR	PR	SD	PD	Toxicity	Morbidity	Mortality
Institution	16.7% (17.4%)	13.3% (15.0%)	30.2% (31.9%)	20.9% (16.7%)	27.2% (10.7%)	12.8% (24.9%)	34.8% (35.2%)	20.8% (14.3%)	22.5% (19.6%)
Study design	1.2% (2.9%)	0.6% (1.6%)	3.1% (1.0%)	10.7% (4.0%)	4.1% (1.8%)	3.6% (6.9%)	3.8% (4.6%)	8.7% (2.0%)	2.3% (1.6%)
Chemotherapy	2.1% (3.1%)	0.8% (1.6%)	0.6% (8.1%)	6.0% (7.2%)	1.6% (0.8%)	0.2% (0.4%)	10.4% (2.4%)	0.1% (0.5%)	3.1% (6.8%)
Mean year of study interval (decades)	2.0% (1.9%)	1.1% (0.2%)	0.2% (2.0%)	0.6% (6.7%)	2.3% (1.0%)	0.9% (7.0%)	2.2% (1.5%)	3.2% (2.0%)	10.4% (10.6%)
Mean patient age (5-y intervals)	1.1% (1.1%)	1.2% (0.6%)	2.7% (1.1%)	3.4% (3.4%)	1.5% (1.8%)	1.4% (1.7%)	3.5% (2.6%)	9.9% (4.1%)	9.9% (4.6%)
Response evaluation criteria			1.5% (3.4%)	2.7% (3.6%)	2.4% (0.1%)	2.6% (0.3%)			
Resectability	14.0% (9.0%)	25.5% (10.5%)	1.0% (0.9%)	0.0% (2.6%)	4.1% (0.9%)	1.1% (8.1%)	0.3% (0.2%)	2.2% (4.9%)	0.6% (0.2%)
Resectability evaluation criteria	0.1% (0.7%)	1.4% (0.7%)							

The fraction of explained variance is given in %. Values in parentheses represent the fraction of explained variance in % from univariable analysis.

CR, complete response; PD, progressive disease; PR, partial response; SD, stable disease.

**Table 8:** Quality assessment.

Category	Outcome							
	Resection Rate	Exploration Rate	Response Evaluation	Toxicity	Morbidity	Mortality	Median Survival	1 y/2 y Survival
Number of studies (range)	35–111	32–88	2–82	22–63	22–50	30–85	19–70	11–54
Study design	Phase I–II trials	Phase I–II trials	Phase I–II trials	Phase I–II trials	Phase I–II trials	Phase I–II trials	Phase I–II trials	Phase I–II trials
	Cohort studies	Cohort studies	Cohort studies	Cohort studies	Cohort studies	Cohort studies	Cohort studies	Cohort studies
	Case series	Case series	Case series	Case series	Case series	Case series	Case series	Case series
Limitations	No serious limitation	No serious limitation	No serious limitation^a^	No serious limitation	No serious limitation^b^	No serious limitation^b^	No serious limitation^c^	No serious limitation^d^
Inconsistency	Serious inconsistency^e^	Serious inconsistency^e^	Serious inconsistency^e^	No serious inconsistency^f^	No serious inconsistency^g^	No serious inconsistency^h^	Serious inconsistency^e^	Serious inconsistency^e^
*Indirectness* i								
Imprecision^j^	No serious imprecision^k^	No serious imprecision^k^	No serious imprecision^k^	No serious imprecision^l^	Serious imprecision^m^	Serious imprecision^n^	Serious imprecision^o^	Serious imprecision^o^
Other considerations							Studies weighted by number of initial patients	Studies weighted by number of initial patients
Quality	++	++	++	+++	++	++	+	+
Importance	Critically important	Important	Important	Important	Important	Critically important	Critically important	Critically important

The quality assessment for the indicated outcome parameters was carried out according to the grade profiler as described in the *Methods* section. +, very low; ++, low; +++, moderate.

a Separated evaluation of response categories, several evaluation criteria, proportions experiencing each type of response are not independent.

b Based only on resected patients.

c Assumption of uniform (exponential) distributed survival times.

d Different trials for estimating 1 y/2 y survival.

e High heterogeneity which could not be sufficiently explained by potential sources of variation within the meta-regression analysis.

f Obvious heterogeneity partly explained by different chemotherapy treatment regimes within the trials.

g Obvious heterogeneity partly explained by differences in mean patient age and study design between the trials.

h No considerable heterogeneity obvious from the data.

i Since no direct comparison was feasible for any considered outcome measurement, indirectness has to be fixed as “very serious” for the entire topic of the investigation.

j For the same reason no particular measure of precision (e.g. confidence intervals) was available for any single trial considered; therefore, imprecision is rather crude assessed in reference to the number of study participants within any meta-analysis.

k Substantial sample sizes per study: median (IQR): 30 (18 to 47).

l Substantial sample sizes per study: median (IQR): 29 (19 to 39).

m Insufficient sample sizes per study: median (IQR): 16 (7 to 25).

n Insufficient sample sizes per study: median (IQR): 13 (6 to 25).

o No sufficient information about patients at risk.

## Discussion

This comprehensive review of neoadjuvant therapy in pancreatic cancer aimed to evaluate the key issues, including aspects of response and survival, and to highlight current problems and drawbacks. Neoadjuvant protocols have been analyzed with increasing frequency (Figure 1), as they offer a number of hypothetical advantages over adjuvant (postoperative) therapy, such as shorter therapy and higher therapy completion rates, tumor down-staging with higher (R0) resection rates, and importantly better patient selection. Thus, neoadjuvant treatment and reassessment may identify those patients (both initially resectable and non-resectable) presenting with rapid progressive or disseminated disease at restaging who therefore have a very poor prognosis and for whom surgery is unlikely to provide any benefit. On the other hand, there is the potential risk for tumor progression during neoadjuvant therapy, i.e. patients with initially resectable tumors might present with local or distant tumor progression at restaging, which might not have occurred in the setting of an initial tumor resection. In addition, neoadjuvant treatment protocols usually require histological confirmation before initiation of therapy, resulting in additional invasive diagnostic measures. Clearly, only randomized controlled trials can clarify which of the hypothetical advantages/disadvantages are real and which ones are not.

There is only one phase III randomized controlled trial being carried out comparing neoadjuvant therapy and surgery with surgery alone (NCT00335543) [38]. This multicenter trial has been recruiting patients since June 2003 and has currently enrolled less than a third of the originally planned 254 patients. Due to the exceedingly slow recruitment, the study will be terminated before reaching the target population.

In the future, phase III trials have to be carried out using already established protocols comparing neoadjuvant therapy followed by exploration and possibly resection, with immediate exploration and resection if possible (and additional standard palliative or adjuvant therapies in both arms). As our data point out, this would be especially relevant in the group of borderline resectable/unresectable tumors. As a prerequisite for such trials, standard definitions of resectability and objective computed tomography criteria should be applied.

The reasons why no other phase III randomized trials for neoadjuvant therapy in pancreatic cancer have been carried out or are currently recruiting patients is not known. It might be speculated that patient recruitment is a problem. However, given the high rate of “neoadjuvant” treated patients with locally advanced/unresectable tumors [6], this argument does not seem to be valid, at least not in this group of patients. Another important problem might be the difficulty to achieve a histological/cytological proof of the tumor; however, this would also apply to palliative therapy in most cases. Obviously, there is a plethora of different chemotherapeutic/radiotherapeutic regimens being used in the neoadjuvant setting (Figure 2, Table S1), and it might be difficult to agree on a specific protocol for a large multi-institutional study. In addition, standardized and widely accepted definitions of resectability criteria are lacking. And finally, it might also be more tempting in terms of funding and publications to perform small phase I–II trials with newer chemotherapeutic agents and radiation protocols, instead of phase III trials with already established protocols. In contrast to the lack of phase III randomized controlled trials, we have identified 111 relevant studies, including 56 phase I–II trials of neoadjuvant therapy in pancreatic cancer.

### Neoadjuvant Therapy for Resectable Pancreatic Cancer

In the group of patients deemed resectable before neoadjuvant treatment, 88.1% of the patients were explored after restaging and of those 85.7% were resected. In all, 73.6% of the patients who were judged resectable were resected after neoadjuvant treatment. This rate is similar to published resection rates of 78%–96% in patients with resectable tumors that are explored without neoadjuvant treatment [4],[39]. Grade 3/4 toxicities observed for neoadjuvant therapy (i.e. radiochemotherapy in 96.4%) were higher than the reported rates for adjuvant chemotherapy but within the range of adjuvant radiochemotherapy. An estimated median survival of 23.3 mo was observed for the group of resectable tumor patients who were resected after treatment. This is within the range of the median survival of 20.1–23.6 mo observed in patients who are resected followed by adjuvant chemotherapy [20],[21],[40], and longer than the median survival of 16.9–20.2 mo for patients who do not receive adjuvant therapy (Figure 7) [20],[21]. In conclusion, the available evidence for resectable pancreatic cancer points to similar resection rates with or without neoadjuvant therapy and similar survival rates comparing neoadjuvant therapy followed by resection versus resection followed by adjuvant therapy (Figure 7).

### Neoadjuvant Therapy for Non-resectable Pancreatic Cancer

In our analysis 46.9% of the patients initially staged unresectable underwent surgical exploration. Of them, 69.9% could be resected successfully, leading to a resectability rate after neoadjuvant treatment in this group of patients of a relevant 33.2% (with comparable R0 resection rates as in the group of initially resectable tumor patients). Morbidity and mortality rates following resection were estimated higher in this group of patients as compared to initially resectable tumor patients, most likely reflecting a more extensive/aggressive surgical approach [41], rather than effects of neoadjuvant therapy. For the group of patients who present with locally advanced/unresectable disease, the median survival is 6–11 mo [42],[43]. Similarly, in our analysis, patients initially diagnosed as unresectable who were not resected had a median survival of 10.2 mo. In contrast, the 33.2% resected patients of the initially non-resectable tumor patients had an estimated median survival of 20.5 mo, which is within the range of pancreatic cancer patients with primary resection and adjuvant therapy. Patients who respond to chemotherapy have a better prognosis than those who do not. Therefore, one can only speculate about the survival time in responding patients if they were not resected. However, the fact that this subgroup of responding patients has the same median survival as patients who underwent immediate resection suggests that the increase in survival time for these patients can probably be attributed to the better treatment (resection) and is not due to patient selection. In conclusion, a relevant proportion, i.e. approximately one third of patients initially staged as locally advanced/unresectable, can be successfully resected following neoadjuvant therapy with an estimated median survival within the range of initially resectable tumor patients (Figure 7).

### Response to Neoadjuvant Therapy

For the whole study cohort the number of patients with complete response was 3.9% and partial response 29.1%. Thus, approximately one third of the patients demonstrate radiographic and/or histological response towards neoadjuvant therapy. These response rates are relatively higher compared to data from palliative chemotherapies (5.5%–14.5% response rate [3]), but similar to published reports on combination chemotherapy (26.8% response rate [44]). Stable disease was observed in 43.9% of the patients, but progressive disease was detected in 20.8%. Interestingly, the data did not differ much in the two groups of initially resectable and non-resectable tumor patients, suggesting similar tumor biology. Future trials will have to address response prediction to identify the approximately one fifth of patients who apparently have a different (more aggressive) tumor biology. Interestingly, an analysis of trials with respect to monotherapy versus combination chemotherapy revealed higher complete and partial response rates in the combination therapy group. Higher response rates, however, did not translate into higher resection rates in the group of initially resectable tumor patients for mono- versus combination therapy. In contrast, the combination therapy resulted in an estimated 20% increase in the resection rate for initially non-resectable tumor patients.

Inherently, a review based on retrospective and prospective phase I–II trials, cohort studies, and case series has several drawbacks:

#### Statistical considerations

Since no data from controlled randomized trials were existent, comparison of subgroups could solely be performed in a descriptive way. Although confidence limits were reported for point estimates of primary interesting proportions (frequency of exploration and/or resection), no effect sizes allowing for direct group comparisons were calculable. By estimating median survival times of study populations, two critical assumptions had to be made: a constant (time-independent) hazard rate and a similar underlying mechanism of patient drop-out (censoring (rates) due to lost follow-up or competing risks) within the trials. Further, no limits of confidence could be provided for estimated median survival times, and thus the presented estimates of median have to be cautiously interpreted as crude estimates of central tendency. Estimates of 1- and 2-y survival were provided as weighted averages. In this term, the total number of study patients was used for weighting because no sufficient information about patients at risk was available. These estimates again assume similar censoring rates within the studies. Further, because several studies had to be used for estimation of survival probabilities, differences between these estimates do not necessarily reflect the real change of survived individuals within one and the same population about time. Frequencies of resection and exploration showed a high heterogeneity (I^2^ values>80%; see below) between the trials. Consequently, although conservative random effect models were used throughout for calculation, point estimates may not reflect underlying latent varieties between the trials and may rather be an artificial average and therefore confidence intervals have to be particularly considered for interpretation.

#### Heterogeneity

Dealing with heterogeneity among study results is one of the most important challenges in meta-analysis. This problem can be partly overcome by the use of random effect models which consider within-study and between-study variability, as well as by stratified analysis of homogeneous study subgroups. Further, meta-regression analyses can be used for explanation of heterogeneity in terms of study-level covariates. In this analysis we have used random effect models and carried out meta-regression analyses to assess sources of heterogeneity. Institutions constituted an important source of heterogeneity in our analysis, underscoring the role of individual (center-specific) approaches/therapy algorithms even in high volume centers of pancreatic surgery that are thought to have comparable outcome parameters [4]. The age of the patients as well as the study period were identified as important variables for heterogeneity especially for perioperative morbidity and in-hospital mortality. Interestingly, differences in the study design (e.g. phase I–II clinical trials, case series, retrospective, prospective studies) had only minor impact on the variability of resection/exploration rates but were an important source of heterogeneity for response evaluation and morbidity.

#### Overlapping patient populations

There were 73 studies from the 17 centers that published more than 1 report with probably partially overlapping patient populations. Some patients might have been included, e.g. in a prospective analysis, later in a comparative analysis or in a retrospective study. Thus, there was the risk of producing artificially precise estimates since the same data were potentially tested multiple times (double counting). However, since the overlapping studies often analyzed different outcome parameters (e.g. toxicity evaluation, pathological response evaluation, etc.), we assumed this risk minor and opted to include these partially overlapping studies. Second, since a large number of outcomes were tested for the same population (multiplicity), there was the risk of false estimates for some of these outcomes. However, since only some studies overlapped, and mostly only by a subset of their populations, we assumed the risk of multiplicity moderate.

#### Definition of resectability

Resectability criteria and especially definitions of borderline resectable/unresectable tumors were variable. Thus, in more than 50% of the studies resectability criteria were not or not clearly stated, thereby constituting a potential source of bias. To minimize these effects we grouped borderline resectable and unresectable tumors together (termed non-resectable tumors), since the definition of resectable tumors is more reliable than the differentiation of borderline resectable and unresectable.

### Conclusion

The present analysis provides the most comprehensive review regarding neoadjuvant therapies in resectable and non-resectable pancreatic cancers to date—thus, the best actual available evidence for response rates, treatment toxicities, resection rates, morbidity and mortality, and survival estimates. The most important findings are that in the group of resectable tumor patients, resection and survival rates after neoadjuvant therapy are similar to the ones observed in primarily resected tumor that are treated by adjuvant therapy. Thus, in this group of patients, the current data do not point to an obvious advantage of neoadjuvant therapy. In contrast, in patients initially staged locally advanced/unresectable, approximately one third of the patients can be resected following neoadjuvant therapy with comparable survival rates as patients who were staged as resectable before treatment. Due to the heterogeneity of applied protocols, data regarding the optimal chemotherapeutic and radiotherapeutic regimen cannot be extrapolated; however, the data suggest that combination chemotherapies result in higher response rates, which is reflected by higher resection rates at least in the group of initially non-resectable tumor patients. Future trials have first to clearly establish the role of neoadjuvant therapy specifically in locally advanced/unresectable tumors and subsequently to define optimal treatment protocols. In addition, common definitions for resectability/non-resectability as well as for response evaluation should be applied. As of now, the available data strongly suggest that patients with locally advanced/unresectable tumors should be included in neoadjuvant protocols and subsequently be re-evaluated for resection, which is possible in a relevant number of patients.

## Supporting Information

Figure S1 PRISMA flow diagram.
(0.07 MB DOC)

Table S1 Summary of the analyzed trials.
(0.60 MB DOC)

Table S2 Currently ongoing/recruiting trials for resectable or non-resectable but non-metastatic PDAC.
(0.07 MB DOC)

Text S1 PRISMA checklist.
(0.07 MB DOC)

Text S2 Review protocol.
(0.04 MB DOC)

## Abbreviations

IORT: intraoperative radiation
IQR: interquartile range
NCCN: National Comprehensive Cancer Network
PDAC: pancreatic ductal adenocarcinoma
