# Supplementary material for: Preoperative/Neoadjuvant Therapy in Pancreatic Cancer: A Systematic Review and Meta-analysis of Response and Resection Percentages
Source: PLoS Med. 2010 Apr 20;7(4):e1000267. doi: 10.1371/journal.pmed.1000267 (PMC2857873; doi:10.1371/journal.pmed.1000267)
Supplement: Figure S1 — PRISMA flow diagram. (0.07 MB DOC) [file pmed.1000267.s001.doc]

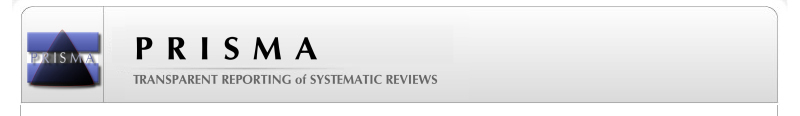
**PRISMA 2009 Flow Diagram**

**Screening**

**Included**

**Eligibility**

**Identification**

Records identified through database searching
(n = 1068)

Additional records identified through other sources
(n = 22)

Records after duplicates removed
(n = 721)

Records screened
(n = 515)

Records excluded
(n = 350)

Articles assessed for eligibility
(n = 165)

Articles excluded

(n = 54)

- *no patients surgically explored/resected*
- *only immunotherapy, no neoadjuvant* *RCT*
- *same patient cohort*

Studies included in qualitative synthesis
(n = 111)

Studies included in quantitative synthesis (meta-analysis)
(n = 111)
