# Supplementary material for: Preoperative/Neoadjuvant Therapy in Pancreatic Cancer: A Systematic Review and Meta-analysis of Response and Resection Percentages
Source: PLoS Med. 2010 Apr 20;7(4):e1000267. doi: 10.1371/journal.pmed.1000267 (PMC2857873; doi:10.1371/journal.pmed.1000267)
Supplement: Table S1 — Summary of the analyzed trials. (0.60 MB DOC) [file pmed.1000267.s002.doc]

**Table S1**: Summary of the analyzed trials

| **First author** | Institution* | Journal | Publication year | Study period | study design^§^ | Prospective/Retrospective | Tumor^$^ | Resectability criteria^#^ | Resectable before neoadjuvant treatment^##^ | Chemotherapy | Radiation dose (in Gy) | Radiation dose (in Gy/fraction) | Age (median) | **Patients CRT and restaging** | **Patients resected** | Morbidity (resected) | Mortality (resected) in hospital | Response criteria^&^ |
| --- | --- | --- | --- | --- | --- | --- | --- | --- | --- | --- | --- | --- | --- | --- | --- | --- | --- | --- |
| **Pilepich MV [1]** | 16 | Cancer | 1980 | 1972-1977 | 5 | P | pc | 3 | 2 | no | 40-50 | 2 Gy/f | 60 | **17** | **6** | 2 | 1 | 3 |
| **Kopelson G [2]** | 16 | Int J Rad Oncol Biol Phys | 1983 | 1972-1981 | 5 | P | pc | 3 | 1 | no | 40-45 | 2 Gy/f | 57 | **5** | **5** | 2 | 0 | 3 |
| **Ishikawa O [3]** | 14 | J Surg Oncol | 1989 | ns | 5 | R | pc+ | 3 | 1 | no | 50 | 2 Gy/f | 65 | **18** | **16** | 2 | 0 | 3 |
| **Weese JL [4]** | 8 | Int J Pancreatol | 1990 | 1986-1989 | 2 | P | pc+ | 3 | 1 | 5-FU/MMC | 50.4 | 1.8 Gy/f | 57 | **16** | **10** | 5 | 2 | 3 |
| **Evans DB [5]** | 12 | Arch Surg | 1992 | 1988-1991 | 1 | P | pc | 3 | 1 | 5-FU | 50.4 | 1.8 Gy/f | 61 | **28** | **17** | 4 | 1 | 2 |
| **Hoffman JP [6]** | 8 | Am Surg | 1993 | 1986-1992 | 5 | R | pc | 3 | 3 | 5-FU/MMC | 50.4 | 1.8 Gy/f | 65 | **39** | **17** | 10 | 2 | 3 |
| **Jessup JM [7]** |  | Arch Surg | 1993 | 1990-1991 | 3 | P | pc | 3 | 2 | 5-FU | 45 | ns | 62 | **16** | **2** | ns | 0 | 3 |
| **Coia L [8]** | 8 | Int J Rad Oncol Biol Phys | 1994 | 1986-1992 | 2 | P | pc+ | 3 | 2 | 5-FU/MMC | 50.4 | 1.8 Gy/f | 64 | **31** | **17** | 8 | 2 | 3 |
| **Ishikawa O [9]** | 14 | Arch Surg | 1994 | 1985-1989 | 4 | P | pc | 3 | 1 | no | 50 | 2 Gy/f | 60 | **23** | **17** | 6 | 0 | 3 |
| **Hoffman JP [10]** | 8 | Am J Surg | 1995 | 1986-1993 | 5 | R | pc | 3 | 3 | 5-FU/MMC | 50.4 | 1.8 Gy/f | 63 | **34** | **13** | 5 | 2 | 2 |
| **Staley CA [11]** | 12 | Am J Surg | 1996 | 1988-1994 | 5 | P | pc+ | 3 | 1 | 5-FU | 30 or 50.4 | ns | ns | **39** | **39** | ns | 1 | 2 |
| **Willet CG [12]** |  | Am J Surg | 1996 | 1990-1994 | 5 | P | pc | 3 | 3 | 5-FU | 45-50.4 | 1.8 Gy/f | 61 | **45** | **9** | ns | ns | 3 |
| **Kamthan AG [13]** |  | J Clin Oncol | 1997 | 1985-1994 | 1 | P | pc | 2 | 2 | 5-FU/Cispl/Streptozocin | 54 | 2 Gy/f | 63 | **35** | **5** | ns | 0 | 2 |
| **Safran H [14]** | 2 | J Clin Oncol | 1997 | 1994-1996 | 1 | P | pc | 3 | 2 | Paclitaxel | 50 | 1.8 Gy/f | 70 | **13** | **1** | ns | ns | 2 |
| **Spitz FR [15]** | 12 | J Clin Oncol | 1997 | 1990-1995 | 5 | P | pc | 2 | 1 | 5-FU | 30 or 50.4 | 1.8 or 3 Gy/f | ns | **91** | **52** | 10 | 0 | 3 |
| **Bousquet J [16]** |  | Chirurgie | 1998 | ns | 5 | P | pc | 2 | 2 | 5-FU/Cispl | 50 | 1.8 Gy/f | 62 | **7** | **2** | 0 | 0 | 3 |
| **Hoffman JP [17]** |  | J Clin Oncol | 1998 | 1991-1993 | 3 | P | pc | 2 | 1 | 5-FU/MMC | 50.4 | 1.8 Gy/f | 65 | **53** | **24** | 3 | 1 | 2 |
| **Pendurthi TK [18]** | 8 | Am Surg | 1998 | 1987-1993 | 4 | R | pc | 3 | 1 | 5-FU/MMC | 50.4 | 1.8 Gy/f | 65 | **25** | **25** | 6 | 1 | 3 |
| **Pisters PW [19]** | 12 | J Clin Oncol | 1998 | ns | 1 | P | pc | 2 | 1 | 5-FU | 30 | 3 Gy/f | ns | **35** | **20** | 11 | 0 | 2 |
| **Todd KE [20]** |  | J Gastrointest Surg | 1998 | 1991-1994 | 1 | P | pc | 3 | 2 | 5-FU/MMC | no | no | 65 | **38** | **4** | 0 | 0 | 2 |
| **Bajetta E [21]** |  | Int J Rad Oncol Biol Phys | 1999 | 1992-1997 | 2 | P | pc | 2 | 2 | Doxifluridine | 50 | 2 Gy/f | 63 | **32** | **5** | ns | ns | 3 |
| **Safran H [22]** | 2 | Semin Radiat Oncol | 1999 | ns | 3 | P | pc+ | 3 | 2 | Paclitaxel | 50 | 1.8 Gy/f | 65 | **18** | **2** | ns | ns | 2 |
| **White RR [23]** | 6 | Ann Surg Oncol | 1999 | 1995-1997 | 5 | R | pc | 2 | 2 | 5-FU and/or MMC/Cispl | 45 | 1.8 Gy/f | 63 | **22** | **5** | 0 | 0 | 3 |
| **Chao C [24]** | 8 | Am Surg | 2000 | 1987-1999 | 4 | R | pc | 3 | 1 | Gem or 5-FU/MMC or Paclitaxel | 50.4 | 1.8 Gy/f | ns | **38** | **20** | 8 | 0 | 2 |
| **Kastl S [25]** | 7 | Eur J Surg Oncol | 2000 | 1995-1997 | 1 | P | pc | 2 | 2 | 5-FU/MMC | 55.8 | 1.8 Gy/f | 61 | **27** | **10** | 4 | 2 | 3 |
| **Kornek GV [26]** | 17 | Br J Cancer | 2000 | 1994-1996 | 2 | P | pc | 3 | 2 | 5-FU/Cispl | 55 | 1.8 Gy/f | 60 | **38** | **3** | ns | 0 | 2 |
| **Snady H [27]** |  | Cancer | 2000 | 1987-1997 | 4 | P | pc | 2 | 2 | 5-FU/Cispl/Streptozocin | 54 | 2 Gy/f | 64 | **68** | **20** | 6 | 2 | 2 |
| **Wanebo HJ [28]** |  | Arch Surg | 2000 | 1990-1992 | 1 | P | pc | 3 | 3 | 5-FU/Cispl | 45 | 1.8 Gy/f | 62 | **14** | **9** | 9 | 1 | 2 |
| **Breslin TM [29]** | 12 | Ann Surg Oncol | 2001 | 1990-1999 | 5 | R | pc | 2 | 1 | 5-FU or Gem or Paclitaxel | 30 or 50.4 | 1.8-3 Gy/f | 62 | **132** | **132** | ns | 2 | 2 |
| **Crane CH [30]** | 12 | Int J Pancreatol | 2001 | 1996-2000 | 2 | P | pc | 2 | 2 | Gem | 30 or 33 | 3 Gy/f | 60 | **51** | **6** | ns | 0 | 3 |
| **Mehta VK [31]** |  | J Gastrointest Surg | 2001 | ns | 5 | P | pc | 2 | 2 | 5-FU | 50.4-56 | 1.8-2 Gy/f | ns | **15** | **9** | 1 | 0 | 2 |
| **Osti MF [32]** | 15 | Tumori | 2001 | 1992-1999 | 3 | P | pc | 3 | 2 | 5-FU | 63 | 1.8 Gy/f | 51 | **31** | **4** | ns | ns | 3 |
| **Pingpank JF [33]** | 8 | J Gastrointest Surg | 2001 | 1987-2000 | 4 | R | pc | 3 | 2 | 5-FU/MMC or Gem | 50.4 | 1.8 Gy/f | 67 | **53** | **53** | 20 | 2 | 3 |
| **Pipas JM [34]** | 5 | Int J Rad Oncol Biol Phys | 2001 | ns | 1 | P | pc | 2 | 3 | Gem | 50.4 | 1.8 Gy/f | 64 | **18** | **5** | ns | 0 | 2 |
| **White RR [35]** | 6 | J Gastrointest Surg | 2001 | 1994-2000 | 5 | R | pc | 2 | 3 | 5-FU or Gem | 40.5 | ns | 64 | **103** | **38** | ns | ns | 3 |
| **White RR [36]** | 6 | Ann Surg Oncol | 2001 | 1995-2000 | 5 | R | pc | 2 | 3 | 5-FU or 5-FU/MMC or 5-FU/MMC/Cispl | 45 | 1.8 Gy/f | 64 | **106** | **39** | 17 | 2 | 3 |
| **Wolff RA [37]** | 12 | Clin Cancer Research | 2001 | 1996-1998 | 1 | P | pc | 2 | 2 | Gem | 30 | 3 Gy/f | 62 | **17** | **1** | 0 | 0 | 2 |
| **Arnoletti JP [38]** | 8 | Am Surg | 2002 | 1988-2001 | 5 | R | pc | 3 | 3 | 5-FU/MMC or Gem | 50.4 | 1.8 Gy/f | 64 | **26** | **10** | ns | 0 | 3 |
| **Crane CH [39]** | 12 | Int J Rad Oncol Biol Phys | 2002 | 1996-2000 | 4 | R | pc | 2 | 2 | 5-FU or Gem | 30-33 | 3 Gy/f | 64 | **114** | **6** | ns | 0 | 2 |
| **deLange SM [40]** |  | Eur J Cancer | 2002 | 1996-1999 | 1 | P | pc | 3 | 2 | Gem | 24 | 8 GY/f | 63 | **24** | **1** | ns | 0 | 2 |
| **Epelbaum R [41]** |  | J Surg Oncol | 2002 | 1997-1999 | 3 | P | pc | 3 | 2 | Gem | 50.4 | 1.8 Gy/f | 66 | **10** | **3** | ns | 0 | 3 |
| **Kim HJ [42]** |  | J Gastrointest Surg | 2002 | 1993-1999 | 5 | R | pc | 3 | 2 | multiple | yes | ns | 66 | **87** | **1** | ns | 0 | 3 |
| **Moutardier V [43]** | 11 | Eur J Surg Oncol | 2002 | 1996-2000 | 3 | P | pc | 3 | 1 | 5-FU/Cispl | 30 or 45 | 1.8 Gy/f | 65 | **19** | **15** | 5 | 0 | 3 |
| **Pisters PW [44]** | 12 | J Clin Oncol | 2002 | ns | 2 | P | pc | 2 | 1 | Paclitaxel | 30 | 3 Gy/f | ns | **35** | **20** | 5 | 0 | 2 |
| **Rau HG [45]** | 9 | Chirurg | 2002 | 1998-2000 | 3 | P | pc | 2 | 2 | Gem/Cispl or Gem/5-FU | 45 | 1.8 Gy/f | 62 | **26** | **14** | 5 | 1 | 3 |
| **Al-Sukhun S [46]** |  | Am J Clin Oncol | 2003 | 1996-1999 | 3 | P | pc | 3 | 2 | 5-FU/Cispl/Cytarabine/Caffeine | 50.4 | 1.8 Gy/f | 59 | **20** | **3** | ns | 0 | 2 |
| **Ammori JB [47]** | 13 | J Gastrointest Surg | 2003 | 1996-2001 | 5 | R | pc | 1 | 2 | Gem or Cispl | 24-50.4 | 1.6-2.4 Gy/f | 63 | **67** | **9** | 1 | 0 | 2 |
| **Aristu J [48]** |  | Am J Clin Oncol | 2003 | 1991-1998 | 5 | R | pc | 2 | 2 | 5-FU/Cispl or Gem/Docetaxel | 45 | 1.8 Gy/f | 63 | **47** | **9** | 8 | 2 | 2 |
| **Brunner TB [49]** | 7 | Int J Rad Oncol Biol Phys | 2003 | 1998-2000 | 1 | P | pc+ | 2 | 2 | Gem/Cispl | 55.8 | 1.8 Gy/f | 61 | **28** | **10** | ns | 0 | 2 |
| **Magnin V [50]** | 11 | Int J Rad Oncol Biol Phys | 2003 | 1996-2001 | 5 | P | pc | 2 | 1 | 5-FU/Cispl | 30-45 | 1.5-1.8 Gy/f | 62 | **32** | **19** | ns | 1 | 3 |
| **Sasson AR [51]** | 8 | Int J Gastrointest Cancer | 2003 | 1987-2000 | 5 | R | pc | 3 | 3 | 5-FU/MMC or Gem | 50.4 | 1.8 Gy/f | 68 | **61** | **61** | ns | ns | 3 |
| **Wilkowski R [52]** | 9 | Strahlenther Onkol | 2003 | 2000-2001 | 4 | P | pc | 2 | 2 | Gem/Cispl | 45 or 50 | 1.8-2 Gy/f | 61 | **33** | **14** | ns | ns | 2 |
| **Calvo FA [53]** |  | Am J Clin Oncol | 2004 | 1998-2001 | 2 | P | pc | 2 | 1 | Tegafur | 45-50.4 | 1.8 Gy/f | 61 | **15** | **9** | ns | ns | 3 |
| **Gnant M [54]** | 17 | 2004 ASCO Annual Meeting | 2004 | ns | 3 | P | pc | 3 | 2 | Gem/Docetaxel | no | no | ns | **61** | **48** | ns | 0 | 3 |
| **Joensuu TK [55]** | 10 | Int J Rad Oncol Biol Phys | 2004 | 1999-2001 | 2 | P | pc | 3 | 1 | Gem | 50.4 | 1.8 Gy/f | 67 | **28** | **20** | ns | ns | 2 |
| **Moutardier V [56]** | 11 | Int J Rad Oncol Biol Phys | 2004 | 1996-2003 | 5 | P | pc | 3 | 1 | 5-FU/Cispl | 45 or 60 | 1.8 or 3 Gy/f | 65 | **61** | **40** | 6 | 2 | 3 |
| **Staley CA [57]** |  | Surg Oncol Clin N Am | 2004 | 1997-2000 | 1 | P | pc+ | 3 | 2 | 5-FU/Cispl/Gem | no | no | 57 | **22** | **1** | ns | 0 | 2 |
| **Wilkowski R [58]** | 9 | World J Surg | 2004 | 2000-2002 | 5 | R | pc | 3 | 2 | Gem/Cispl | 45 or 50 | 1.8-2 Gy/f | 61 | **45** | **20** | ns | ns | 2 |
| **Zimmermann FB [59]** |  | Hepatogastroenterology | 2004 | 1999-2000 | 5 | P | pc | 3 | 2 | 5-FU or Cispl/Gem | 30 | 3 Gy/f | 59 | **17** | **11** | ns | 0 | 2 |
| **Bettini N [60]** | 11 | Gastroenterol Clin Biol | 2005 | 1996-2003 | 3 | P | pc | 2 | 1 | 5-FU/Cispl | 30 or 45 | 1.8 or 3 Gy/f | 61 | **45** | **30** | ns | 0 | 3 |
| **Magnino A [61]** | 3 | Oncology | 2005 | 1999-2003 | 3 | P | pc | 2 | 2 | Gem | 45 | 1.8 Gy/f | 62 | **18** | **6** | ns | 1 | 1 |
| **Ohigashi H [62]** | 14 | Ann Surg Oncol | 2005 | 1995-2002 | 3 | P | pc | 3 | 2 | 5-FU | 24 | 2 Gy/f | 56 | **19** | **15** | 3 | 0 | 2 |
| **Pipas JM [63]** | 5 | Ann Surg Oncol | 2005 | 2002-2004 | 3 | P | pc | 2 | 3 | Gem/Docetaxel | 50.4 | 1.8 Gy/f | 65 | **24** | **17** | ns | 2 | 2 |
| **Sa Cunha A [64]** | 1 | Am Coll Surg | 2005 | 1998-2003 | 5 | R | pc | 2 | 2 | 5-FU/Cispl | 45 | 1.8 Gy/f | ns | **61** | **13** | 3 | 0 | 3 |
| **Smeenk HG [65]** |  | Dig Surg | 2005 | 1982-1998 | 4 | R | pc | 2 | 2 | 5-FU | 50 | 2 Gy/f | 62 | **38** | **3** | ns | 0 | 2 |
| **White RR [66]** | 6 | Ann Surg Oncol | 2005 | ns | 5 | R | pc | 3 | 3 | 5-FU or Gem | 30-50.4 | 1.5-1.8 Gy/f | ns | **193** | **70** | ns | 3 | 2 |
| **Adhoute X [67]** | 1 | Gastroenterol Clin Biol | 2006 | 1996-2001 | 5 | P | pc | 2 | 2 | 5-FU/Cispl | 45-50 | 1.8 or 2 Gy/f | 63 | **33** | **8** | ns | ns | 2 |
| **Czito BG [68]** | 6 | Cancer Invest | 2006 | 1998-2000 | 1 | P | pc+ | 2 | 3 | 5-FU/Eniluracil | 45 | 1.8 Gy/f | 66 | **13** | **2** | ns | ns | 3 |
| **Delpero JR [69]** | 11 | Cancer/Radiotherapie | 2006 | 1998-2005 | 5 | R | pc | 3 | 2 | 5-FU/Cispl or 5-FU/Docetaxel | 45 | ns | ns | **26** | **15** | 5 | 2 | 3 |
| **Krempien RC [70]** |  | 2006 ASCO Annual Meeting | 2006 | ns | 3 | P | pc | 3 | 2 | Gem/Cetuximab | 54 | ns | 64 | **20** | **4** | ns | ns | 3 |
| **Massucco P [71]** | 3 | Ann Surg Oncol | 2006 | 1999-2003 | 3 | P | pc | 2 | 2 | Gem | 45 | 1.8 Gy/f | 62 | **28** | **8** | 4 | 0 | 1 |
| **Mornex F [72]** |  | Int J Rad Oncol | 2006 | 1998-2003 | 3 | P | pc | 3 | 1 | 5-FU/Cispl | 50 | 2 Gy/f | 59 | **40** | **26** | 13 | 1 | 3 |
| **Talamonti MS [73]** |  | Ann Surg Oncol | 2006 | 2002-2003 | 3 | P | pc | 1 | 1 | Gem | 36 | 2.4 Gy/f | 58 | **20** | **17** | 4 | 0 | 2 |
| **White RR [74]** |  | Ann Surg Oncol | 2006 | 1994-2004 | 4 | R | pc | 3 | 3 | 5-FU and/or MMC and/or Cispl or Gem | 50.4 | 1.8 Gy/f | 62 | **82** | **82** | ns | ns | 3 |
| **Wilkowski R [75]** | 9 | JOP | 2006 | 1999-2006 | 5 | P | pc | 3 | 2 | Gem/5-FU or Gem/Cispl | 45 or 50 | 1.8 or 2 Gy/f | 70 | **32** | **7** | ns | 2 | 2 |
| **Desai SP [76]** | 13 | J Clin Oncol | 2007 | 2004-2006 | 1 | P | pc | 1 | 1 | Gem/Oxalipl | 27-30 | 1.8 Gy/f | 64 | **12** | **7** | ns | 0 | 1 |
| **Fogelman DR [77]** | 4 | 2007 Gastrointestinal Cancers Symposium | 2007 | ns | 3 | P | pc | 3 | 2 | Gem/Capecetabine/Docetaxel | yes | ns | 58 | **14** | **8** | ns | 0 | 1 |
| **Macchia G [78]** | 15 | Tumori | 2007 | ns | 3 | P | pc | 3 | 3 | 5-FU | 39.6 | 1.8 Gy/f | 63 | **28** | **9** | ns | 0 | 2 |
| **Nakamori S [79]** | 14 | 2007 Gastrointestinal Cancers Symposium | 2007 | ns | 1 | P | pc | 3 | 1 | Gem | 30 or 36 | 1.5 Gy/f (H) | ns | **15** | **11** | ns | ns | 3 |
| **Palmer DH [80]** |  | Ann Surg Oncol | 2007 | 1999-2003 | 3 | P | pc | 2 | 1 | Gem or Gem/Cispl | no | no | 66 | **50** | **27** | 3 | 13 | 3 |
| **Takamori H [81]** |  | 2007 ASCO Annual Meeting | 2007 | 2001-2006 | 3 | P | pc | 3 | 1 | Gem | no | ns | ns | **32** | **32** | ns | ns | 3 |
| **Vento P [82]** | 10 | World J Gastroenterol | 2007 | 1999-2002 | 4 | P | pc | 3 | 1 | Gem | 50 | 1.8 Gy/f | 65 | **22** | **22** | 10 | 0 | 3 |
| **Yamazaki H [83]** | 14 | Strahlenther Onkol | 2007 | 2002-2003 | 5 | P | pc | 3 | 3 | Gem | 50 | 2 Gy/f | 66 | **12** | **9** | ns | ns | 3 |
| **Allendorf JD [84]** | 4 | J Gastrointest Surg | 2008 | 2000-2006 | 4 | R | pc | 3 | 2 | Gem/Capecetabine/Docetaxel | 50.4 | 1.8 Gy/f | 60 | **78** | **59** | 26 | 6 | 2 |
| **Brown KM [85]** | 8 | Am J Surg | 2008 | 2004-2007 | 5 | R | pc | 2 | 2 | 5-FU or Gem or Capecitabine/Bevacizumab or 5-FU/Erlotinib | 50.4 | 1.8 Gy/f | 61 | **13** | **13** | 5 | 0 | 3 |
| **Evans DB [86]** | 12 | J Clin Oncol | 2008 | 1998-2001 | 3 | P | pc | 2 | 1 | Gem | 30 | 3 Gy/f | 64 | **86** | **64** | 6 | 1 | 2 |
| **Golcher H [87]** | 7 | Eur J Surg Oncol | 2008 | 1995-2003 | 4 | R | pc | 2 | 1 | 5-FU/MMC | 55.8 | 1.8 Gy/f | 60 | **50** | **21** | 5 | 1 | 3 |
| **Greer SE [88]** | 5 | J Am Coll Surg | 2008 | 1993-2005 | 5 | R | pc | 2 | 3 | Gem or Cispl/5-FU or Gem/Docetaxel | 45 or 50.4 | 1.8 Gy/f | 63 | **42** | **42** | ns | 4 | 3 |
| **Heinrich S [89]** |  | J Clin Oncol | 2008 | 2001-2007 | 3 | P | pc | 3 | 1 | Gem/Cispl | no | ns | 59 | **28** | **25** | ns | 1 | 1 |
| **Le Scodan R [90]** |  | Am J Clin Oncol | 2008 | 1998-2003 | 3 | P | pc | 3 | 1 | Gem/Cispl | 50 | 2 Gy/f | 59 | **41** | **26** | 13 | 1 | 2 |
| **Lind PA [91]** |  | Acta Oncol | 2008 | 2002-2004 | 4 | R | pc | 2 | 2 | Capecetabine/Oxalipl | 50.4 | 1.8 Gy/f | 58 | **17** | **8** | 4 | 0 | 3 |
| **Marti JL [92]** |  | Ann Surg Oncol | 2008 | 1997-2004 | 2 | P | pc | 2 | 2 | Gem or Cispl | 50.4 | 1.8 Gy/f | 63 | **18** | **4** | 3 | 0 | 3 |
| **Small W [93]** |  | 2008 Gastrointestinal Cancers Symposium | 2008 | ns | 3 | P | pc | 3 | 2 | Gem/Bevacizumab | 36 | 2.4 Gy/f | ns | **29** | **5** | ns | ns | 3 |
| **Stitzenberg KB [94]** | 8 | Ann Surg Oncol | 2008 | 1996-2007 | 5 | R | pc | 1 | 2 | 5-FU or Capecitabine or Gem | yes | ns | 62 | **12** | **12** | 12 | 2 | 3 |
| **Takai S [95]** | 14 | Pancreas | 2008 | 2000-2004 | 4 | R | pc | 1 | 1 | 5-FU/Cispl or Gem | 40 | 2 Gy/f | 62 | **31** | **24** | ns | 0 | 2 |
| **Varadhachary GR [96]** | 12 | J Clin Oncol | 2008 | 2002-2006 | 3 | P | pc | 2 | 1 | Gem/Cispl | 30 | 3 Gy/f | 64 | **79** | **52** | 5 | 0 | 2 |
| **Bjerregaard JK [97]** |  | Radiother Oncol | 2009 | 2001-2005 | 5 | P | pc | 3 | 2 | 5-FU | 50 | 1.8 Gy/f | 63 | **54** | **11** | ns | 0 | 3 |
| **Cardenes HR [98]** |  | 2009 Gastrointestinal Cancers Symposium | 2009 | ns | 2 | P | pc | 3 | 1 | Gem/Erlotinib | 30 | 3 Gy/f (H) | 61 | **10** | **6** | ns | 0 | 3 |
| **Chaudhary UB [99]** |  | 2009 Gastrointestinal Cancers Symposium | 2009 | ns | 3 | P | pc | 3 | 2 | Gem/Oxalipl/Cetuximab | 54 | ns | 57 | **32** | **8** | ns | ns | 3 |
| **Chen J [100]** | 12 | Ann Surg Oncol | 2009 | 1999-2004 | 3 | P | pc | 3 | 1 | Gem and/or Cispl | 30 | 3 Gy/f | 65 | **88** | **69** | ns | ns | 3 |
| **Choi M [101]** |  | Am J Clin Oncol | 2009 | ns | 5 | P | pc | 3 | 2 | 5-FU/Cispl/Cytarabine/Caffeine | 39.6 or 50.4 | 1.8 Gy/f | ns | **20** | **3** | ns | 0 | 3 |
| **Kim YE [102]** |  | Radiology | 2009 | 2002-2007 | 5 | R | pc | 2 | 2 | Gem | 45 or 50 | ns | 61 | **12** | **10** | ns | ns | 3 |
| **Laurent S [103]** |  | Ann Oncol | 2009 | ns | 2 | P | pc+ | 3 | 2 | Gem/Oxalipl | 45 | 1.8 Gy/f | 61 | **24** | **4** | ns | ns | 3 |
| **Masui T [104]** |  | 2009 Gastrointestinal Cancers Symposium | 2009 | ns | 2 | P | pc | 2 | 2 | Gem/S1 | no | no | ns | **18** | **12** | ns | ns | 3 |
| **Maximous DW [105]** |  | Int Arch Med | 2009 | 2006-2007 | 5 | P | pc | 3 | 2 | Gem | 54 | 1.8 Gy/f | 46 | **25** | **8** | 5 | 1 | 3 |
| **Ohigashi H [106]** | 14 | Ann Surg | 2009 | 2002-2007 | 2 | P | pc | 2 | 3 | Gem | 50 | 2 Gy/f | 66 | **38** | **31** | 4 | 0 | 1 |
| **Rifkind J [107]** | 5 | 2009 Gastrointestinal Cancers Symposium | 2009 | 1996-2006 | 5 | R | pc | 3 | 3 | Gem | 50.4 | ns | 67 | **113** | **64** | ns | ns | 3 |
| **Satoi S [108]** | 14 | Pancreas | 2009 | 2001-2004 | 5 | P | pc | 1 | 3 | 5-FU/Cispl or Gem | 40 | 2 Gy/f | 64 | **34** | **27** | ns | 0 | 3 |
| **Stokes JB [109]** | 13 | 2009 Gastrointestinal Cancers Symposium | 2009 | 2005-2008 | 5 | R | pc | 2 | 2 | Gem or Capecetabine | 50 | 1.8 or 2.5 Gy/f | ns | **35** | **16** | ns | ns | 3 |
| **Tinkl D [110]** | 7 | Strahlenther Onkol | 2009 | 1996-2006 | 5 | P | pc+ | 1 | 2 | 5-FU/MMC or Gem/Cispl | 55.8 | 1.8 Gy/f | 61 | **120** | **38** | ns | 5 | 2 |
| **Turrini O [111]** | 11 | Oncology | 2009 | 1996-2007 | 5 | R | pc | 2 | 1 | 5-FU/Cispl | 45 | 1.8 Gy/f | ns | **101** | **62** | 11 | 4 | 2 |

* centers with more than one published study (1: Centre Hospitalier Universitaire de Bordeaux, France; 2: Brown University, RI; 3: Institute for Research and Cure of Cancer, Candiolo, Italy; 4: Columbia University New York, NY; 5: Dartmouth Medical Center, NH; 6: Duke University, NC; 7: University of Erlangen, Germany; 8: Fox Chase Cancer Center Philadelphia, PA; 9: University of Munich, Germany; 10: Helsinki University, Finland; 11:  University of Marseille, France; 12: MD Anderson Cancer Center, Houston, TX; 13: Michigan University, Ann Arbor, MI; 14: Osaka University, Japan; 15: Rome University, Italy; 16: Tufts University, MA; 17: Vienna University, Austria)

^§^ 1: phase I trial; 2: phase I/II trial; 3: phase II trial; 4: cohort study; 5: case series

^$^ pc: pancreatic cancer; pc+: pancreatic cancer and other periampullary tumors

^#^ 1: NCCN resectability criteria; 2. well defined resectability criteria; 3: not clearly defined or not stated resectability criteria

^##^ 1: resectable; 2: non-resectable (borderline/unresectable); 3: both resectable and non-resectable

^&^ 1: RECIST response criteria; 2: well defined response criteria; 3: not clearly defined or not stated response criteria

MMC: Mitomycin-C; Cispl: Cisplatin; Gem: Gemcitabine; Oxalipl: Oxaliplatin; 5-FU: 5-Fluorouracil; ns: not stated; Gy/f: Gray/fraction

**References**

1. Pilepich MV, Miller HH (1980) Preoperative irradiation in carcinoma of the pancreas. Cancer 46: 1945-1949.

2. Kopelson G (1983) Curative surgery for adenocarcinoma of the pancreas/ampulla of Vater: the role of adjuvant pre or postoperative radiation therapy. Int J Radiat Oncol Biol Phys 9: 911-915.

3. Ishikawa O, Ohhigashi H, Teshima T, Chatani M, Inoue T, et al. (1989) Clinical and histopathological appraisal of preoperative irradiation for adenocarcinoma of the pancreatoduodenal region. J Surg Oncol 40: 143-151.

4. Weese JL, Nussbaum ML, Paul AR, Engstrom PF, Solin LJ, et al. (1990) Increased resectability of locally advanced pancreatic and periampullary carcinoma with neoadjuvant chemoradiotherapy. Int J Pancreatol 7: 177-185.

5. Evans DB, Rich TA, Byrd DR, Cleary KR, Connelly JH, et al. (1992) Preoperative chemoradiation and pancreaticoduodenectomy for adenocarcinoma of the pancreas. Arch Surg 127: 1335-1339.

6. Hoffman JP, Weese JL, Solin LJ, Agarwal P, Engstrom P, et al. (1993) A single institutional experience with preoperative chemoradiotherapy for stage I-III pancreatic adenocarcinoma. Am Surg 59: 772-780; discussion 780-771.

7. Jessup JM, Steele G, Jr., Mayer RJ, Posner M, Busse P, et al. (1993) Neoadjuvant therapy for unresectable pancreatic adenocarcinoma. Arch Surg 128: 559-564.

8. Coia L, Hoffman J, Scher R, Weese J, Solin L, et al. (1994) Preoperative chemoradiation for adenocarcinoma of the pancreas and duodenum. Int J Radiat Oncol Biol Phys 30: 161-167.

9. Ishikawa O, Ohigashi H, Imaoka S, Sasaki Y, Iwanaga T, et al. (1994) Is the long-term survival rate improved by preoperative irradiation prior to Whipple's procedure for adenocarcinoma of the pancreatic head? Arch Surg 129: 1075-1080.

10. Hoffman JP, Weese JL, Solin LJ, Engstrom P, Agarwal P, et al. (1995) A pilot study of preoperative chemoradiation for patients with localized adenocarcinoma of the pancreas. Am J Surg 169: 71-77; discussion 77-78.

11. Staley CA, Lee JE, Cleary KR, Abbruzzese JL, Fenoglio CJ, et al. (1996) Preoperative chemoradiation, pancreaticoduodenectomy, and intraoperative radiation therapy for adenocarcinoma of the pancreatic head. Am J Surg 171: 118-124; discussion 124-115.

12. Willett CG, Daly WJ, Warshaw AL (1996) CA 19-9 is an index of response to neoadjunctive chemoradiation therapy in pancreatic cancer. Am J Surg 172: 350-352.

13. Kamthan AG, Morris JC, Dalton J, Mandeli JP, Chesser MR, et al. (1997) Combined modality therapy for stage II and stage III pancreatic carcinoma. J Clin Oncol 15: 2920-2927.

14. Safran H, King TP, Choy H, Hesketh PJ, Wolf B, et al. (1997) Paclitaxel and concurrent radiation for locally advanced pancreatic and gastric cancer: a phase I study. J Clin Oncol 15: 901-907.

15. Spitz FR, Abbruzzese JL, Lee JE, Pisters PW, Lowy AM, et al. (1997) Preoperative and postoperative chemoradiation strategies in patients treated with pancreaticoduodenectomy for adenocarcinoma of the pancreas. J Clin Oncol 15: 928-937.

16. Bousquet J, Slim K, Pezet D, Alexandre M, Verrelle P, et al. (1998) [Does neoadjuvant radiochemotherapy augment the resectability of pancreatic cancers?]. Chirurgie 123: 456-460.

17. Hoffman JP, Lipsitz S, Pisansky T, Weese JL, Solin L, et al. (1998) Phase II trial of preoperative radiation therapy and chemotherapy for patients with localized, resectable adenocarcinoma of the pancreas: an Eastern Cooperative Oncology Group Study. J Clin Oncol 16: 317-323.

18. Pendurthi TK, Hoffman JP, Ross E, Johnson DE, Eisenberg BL (1998) Preoperative versus postoperative chemoradiation for patients with resected pancreatic adenocarcinoma. Am Surg 64: 686-692.

19. Pisters PW, Abbruzzese JL, Janjan NA, Cleary KR, Charnsangavej C, et al. (1998) Rapid-fractionation preoperative chemoradiation, pancreaticoduodenectomy, and intraoperative radiation therapy for resectable pancreatic adenocarcinoma. J Clin Oncol 16: 3843-3850.

20. Todd KE, Gloor B, Lane JS, Isacoff WH, Reber HA (1998) Resection of locally advanced pancreatic cancer after downstaging with continuous-infusion 5-fluorouracil, mitomycin-C, leucovorin, and dipyridamole. J Gastrointest Surg 2: 159-166.

21. Bajetta E, Di Bartolomeo M, Stani SC, Artale S, Ricci SB, et al. (1999) Chemoradiotherapy as preoperative treatment in locally advanced unresectable pancreatic cancer patients: results of a feasibility study. Int J Radiat Oncol Biol Phys 45: 285-289.

22. Safran H, Akerman P, Cioffi W, Gaissert H, Joseph P, et al. (1999) Paclitaxel and concurrent radiation therapy for locally advanced adenocarcinomas of the pancreas, stomach, and gastroesophageal junction. Semin Radiat Oncol 9: 53-57.

23. White R, Lee C, Anscher M, Gottfried M, Wolff R, et al. (1999) Preoperative chemoradiation for patients with locally advanced adenocarcinoma of the pancreas. Ann Surg Oncol 6: 38-45.

24. Chao C, Hoffman JP, Ross EA, Torosian MH, Eisenberg BL (2000) Pancreatic carcinoma deemed unresectable at exploration may be resected for cure: an institutional experience. Am Surg 66: 378-385; discussion 386.

25. Kastl S, Brunner T, Herrmann O, Riepl M, Fietkau R, et al. (2000) Neoadjuvant radio-chemotherapy in advanced primarilynon-resectable carcinomas of the pancreas. Eur J Surg Oncol 26: 578-582.

26. Kornek GV, Schratter-Sehn A, Marczell A, Depisch D, Karner J, et al. (2000) Treatment of unresectable, locally advanced pancreatic adenocarcinoma with combined radiochemotherapy with 5-fluorouracil, leucovorin and cisplatin. Br J Cancer 82: 98-103.

27. Snady H, Bruckner H, Cooperman A, Paradiso J, Kiefer L (2000) Survival advantage of combined chemoradiotherapy compared with resection as the initial treatment of patients with regional pancreatic carcinoma. An outcomes trial. Cancer 89: 314-327.

28. Wanebo HJ, Glicksman AS, Vezeridis MP, Clark J, Tibbetts L, et al. (2000) Preoperative chemotherapy, radiotherapy, and surgical resection of locally advanced pancreatic cancer. Arch Surg 135: 81-87; discussion 88.

29. Breslin TM, Hess KR, Harbison DB, Jean ME, Cleary KR, et al. (2001) Neoadjuvant chemoradiotherapy for adenocarcinoma of the pancreas: treatment variables and survival duration. Ann Surg Oncol 8: 123-132.

30. Crane CH, Janjan NA, Evans DB, Wolff RA, Ballo MT, et al. (2001) Toxicity and efficacy of concurrent gemcitabine and radiotherapy for locally advanced pancreatic cancer. Int J Pancreatol 29: 9-18.

31. Mehta VK, Fisher G, Ford JA, Poen JC, Vierra MA, et al. (2001) Preoperative chemoradiation for marginally resectable adenocarcinoma of the pancreas. J Gastrointest Surg 5: 27-35.

32. Osti MF, Costa AM, Bianciardi F, De Nicolo M, Donato V, et al. (2001) Concomitant radiotherapy with protracted 5-fluorouracil infusion in locally advanced carcinoma of the pancreas: a phase II study. Tumori 87: 398-401.

33. Pingpank JF, Hoffman JP, Ross EA, Cooper HS, Meropol NJ, et al. (2001) Effect of preoperative chemoradiotherapy on surgical margin status of resected adenocarcinoma of the head of the pancreas. J Gastrointest Surg 5: 121-130.

34. Pipas JM, Mitchell SE, Barth RJ, Jr., Vera-Gimon R, Rathmann J, et al. (2001) Phase I study of twice-weekly gemcitabine and concomitant external-beam radiotherapy in patients with adenocarcinoma of the pancreas. Int J Radiat Oncol Biol Phys 50: 1317-1322.

35. White RR, Paulson EK, Freed KS, Keogan MT, Hurwitz HI, et al. (2001) Staging of pancreatic cancer before and after neoadjuvant chemoradiation. J Gastrointest Surg 5: 626-633.

36. White RR, Hurwitz HI, Morse MA, Lee C, Anscher MS, et al. (2001) Neoadjuvant chemoradiation for localized adenocarcinoma of the pancreas. Ann Surg Oncol 8: 758-765.

37. Wolff RA, Evans DB, Gravel DM, Lenzi R, Pisters PW, et al. (2001) Phase I trial of gemcitabine combined with radiation for the treatment of locally advanced pancreatic adenocarcinoma. Clin Cancer Res 7: 2246-2253.

38. Arnoletti JP, Hoffman JP, Ross EA, Kagan SA, Meropol NJ, et al. (2002) Preoperative chemoradiation in the management of adenocarcinoma of the body of the pancreas. Am Surg 68: 330-335; discussion 335-336.

39. Crane CH, Abbruzzese JL, Evans DB, Wolff RA, Ballo MT, et al. (2002) Is the therapeutic index better with gemcitabine-based chemoradiation than with 5-fluorouracil-based chemoradiation in locally advanced pancreatic cancer? Int J Radiat Oncol Biol Phys 52: 1293-1302.

40. de Lange SM, van Groeningen CJ, Meijer OW, Cuesta MA, Langendijk JA, et al. (2002) Gemcitabine-radiotherapy in patients with locally advanced pancreatic cancer. Eur J Cancer 38: 1212-1217.

41. Epelbaum R, Rosenblatt E, Nasrallah S, Faraggi D, Gaitini D, et al. (2002) Phase II study of gemcitabine combined with radiation therapy in patients with localized, unresectable pancreatic cancer. J Surg Oncol 81: 138-143.

42. Kim HJ, Czischke K, Brennan MF, Conlon KC (2002) Does neoadjuvant chemoradiation downstage locally advanced pancreatic cancer? J Gastrointest Surg 6: 763-769.

43. Moutardier V, Giovannini M, Lelong B, Monges G, Bardou VJ, et al. (2002) A phase II single institutional experience with preoperative radiochemotherapy in pancreatic adenocarcinoma. Eur J Surg Oncol 28: 531-539.

44. Pisters PW, Wolff RA, Janjan NA, Cleary KR, Charnsangavej C, et al. (2002) Preoperative paclitaxel and concurrent rapid-fractionation radiation for resectable pancreatic adenocarcinoma: toxicities, histologic response rates, and event-free outcome. J Clin Oncol 20: 2537-2544.

45. Rau HG, Wichmann MW, Wilkowski R, Heinemann V, Sackmann M, et al. (2002) [Surgical therapy of locally advanced and primary inoperable pancreatic carcinoma after neoadjuvant preoperative radiochemotherapy]. Chirurg 73: 132-137.

46. Al-Sukhun S, Zalupski MM, Ben-Josef E, Vaitkevicius VK, Philip PA, et al. (2003) Chemoradiotherapy in the treatment of regional pancreatic carcinoma: a phase II study. Am J Clin Oncol 26: 543-549.

47. Ammori JB, Colletti LM, Zalupski MM, Eckhauser FE, Greenson JK, et al. (2003) Surgical resection following radiation therapy with concurrent gemcitabine in patients with previously unresectable adenocarcinoma of the pancreas. J Gastrointest Surg 7: 766-772.

48. Aristu J, Canon R, Pardo F, Martinez-Monge R, Martin-Algarra S, et al. (2003) Surgical resection after preoperative chemoradiotherapy benefits selected patients with unresectable pancreatic cancer. Am J Clin Oncol 26: 30-36.

49. Brunner TB, Grabenbauer GG, Klein P, Baum U, Papadopoulos T, et al. (2003) Phase I trial of strictly time-scheduled gemcitabine and cisplatin with concurrent radiotherapy in patients with locally advanced pancreatic cancer. Int J Radiat Oncol Biol Phys 55: 144-153.

50. Magnin V, Moutardier V, Giovannini MH, Lelong B, Giovannini M, et al. (2003) Neoadjuvant preoperative chemoradiation in patients with pancreatic cancer. Int J Radiat Oncol Biol Phys 55: 1300-1304.

51. Sasson AR, Wetherington RW, Hoffman JP, Ross EA, Cooper H, et al. (2003) Neoadjuvant chemoradiotherapy for adenocarcinoma of the pancreas: analysis of histopathology and outcome. Int J Gastrointest Cancer 34: 121-128.

52. Wilkowski R, Thoma M, Heinemann V, Rau HG, Wagner A, et al. (2003) [Radiochemotherapy with gemcitabine and cisplatin in pancreatic cancer -- feasible and effective]. Strahlenther Onkol 179: 78-86.

53. Calvo FA, Matute R, Garcia-Sabrido JL, Gomez-Espi M, Martinez NE, et al. (2004) Neoadjuvant chemoradiation with tegafur in cancer of the pancreas: initial analysis of clinical tolerance and outcome. Am J Clin Oncol 27: 343-349.

54. Gnant M, Kuehrer I, Teleky B, Goetzinger P, Penz M, et al. (2004) Effect of neoadjuvant chemotherapy with gemcitabine and docetaxel on 3-year survival and resection rate in previously unresectable locally advanced pancreatic cancer. J Clin Oncol 22: 4234.

55. Joensuu TK, Kiviluoto T, Karkkainen P, Vento P, Kivisaari L, et al. (2004) Phase I-II trial of twice-weekly gemcitabine and concomitant irradiation in patients undergoing pancreaticoduodenectomy with extended lymphadenectomy for locally advanced pancreatic cancer. Int J Radiat Oncol Biol Phys 60: 444-452.

56. Moutardier V, Magnin V, Turrini O, Viret F, Hennekinne-Mucci S, et al. (2004) Assessment of pathologic response after preoperative chemoradiotherapy and surgery in pancreatic adenocarcinoma. Int J Radiat Oncol Biol Phys 60: 437-443.

57. Staley CA, Harris WB, Landry J, Small W, Kooby D, et al. (2004) Neoadjuvant induction chemotherapy followed by chemoradiation: a phase I trial of gemcitabine, cisplatin, and 5-fluorouracil for advanced pancreatic/gastrointestinal malignancies. Surg Oncol Clin N Am 13: 697-709, x.

58. Wilkowski R, Thoma M, Schauer R, Wagner A, Heinemann V (2004) Effect of chemoradiotherapy with gemcitabine and cisplatin on locoregional control in patients with primary inoperable pancreatic cancer. World J Surg 28: 1011-1018.

59. Zimmermann FB, Schuhmacher C, Lersch C, Bernhardt B, Pickel P, et al. (2004) Sequential and/or concurrent hypofractionated radiotherapy and concurrent chemotherapy in neoadjuvant treatment of advanced adenocarcinoma of the pancreas. Outcome and patterns of failure. Hepatogastroenterology 51: 1842-1846.

60. Bettini N, Moutardier V, Turrini O, Bories E, Monges G, et al. (2005) Preoperative locoregional re-evaluation by endoscopic ultrasound in pancreatic ductal adenocarcinoma after neoadjuvant chemoradiation. Gastroenterol Clin Biol 29: 659-663.

61. Magnino A, Gatti M, Massucco P, Sperti E, Faggiuolo R, et al. (2005) Phase II trial of primary radiation therapy and concurrent chemotherapy for patients with locally advanced pancreatic cancer. Oncology 68: 493-499.

62. Ohigashi H, Ishikawa O, Eguchi H, Sasaki Y, Yamada T, et al. (2005) Feasibility and efficacy of combination therapy with preoperative and postoperative chemoradiation, extended pancreatectomy, and postoperative liver perfusion chemotherapy for locally advanced cancers of the pancreatic head. Ann Surg Oncol 12: 629-636.

63. Pipas JM, Barth RJ, Jr., Zaki B, Tsapakos MJ, Suriawinata AA, et al. (2005) Docetaxel/Gemcitabine followed by gemcitabine and external beam radiotherapy in patients with pancreatic adenocarcinoma. Ann Surg Oncol 12: 995-1004.

64. Sa Cunha A, Rault A, Laurent C, Adhoute X, Vendrely V, et al. (2005) Surgical resection after radiochemotherapy in patients with unresectable adenocarcinoma of the pancreas. J Am Coll Surg 201: 359-365.

65. Smeenk HG, de Castro SM, Jeekel JJ, Kazemier G, Busch OR, et al. (2005) Locally advanced pancreatic cancer treated with radiation and 5-fluorouracil: a first step to neoadjuvant treatment? Dig Surg 22: 191-197.

66. White RR, Xie HB, Gottfried MR, Czito BG, Hurwitz HI, et al. (2005) Significance of histological response to preoperative chemoradiotherapy for pancreatic cancer. Ann Surg Oncol 12: 214-221.

67. Adhoute X, Smith D, Vendrely V, Rault A, Sa Cunha A, et al. (2006) Subsequent resection of locally advanced pancreatic carcinoma after chemoradiotherapy. Gastroenterol Clin Biol 30: 224-230.

68. Czito BG, Hong TJ, Cohen DP, Petros WP, Tyler DS, et al. (2006) A phase I study of eniluracil/5-FU in combination with radiation therapy for potentially resectable and/or unresectable cancer of the pancreas and distal biliary tract. Cancer Invest 24: 9-17.

69. Delpero JR, Turrini O (2006) [Locally advanced pancreatic adenocarcinoma. Chemoradiotherapy, reevaluation and secondary resection]. Cancer Radiother 10: 462-470.

70. Krempien RC, Münter MW, Timke C, Huber PE, Friess H, et al. (2006) Phase II study evaluating trimodal therapy with cetuximab intensity modulated radiotherapy (IMRT) and gemcitabine for patients with locally advanced pancreatic cancer [ISRCTN56652283]. J Clin Oncol 24: 4100.

71. Massucco P, Capussotti L, Magnino A, Sperti E, Gatti M, et al. (2006) Pancreatic resections after chemoradiotherapy for locally advanced ductal adenocarcinoma: analysis of perioperative outcome and survival. Ann Surg Oncol 13: 1201-1208.

72. Mornex F, Girard N, Scoazec JY, Bossard N, Ychou M, et al. (2006) Feasibility of preoperative combined radiation therapy and chemotherapy with 5-fluorouracil and cisplatin in potentially resectable pancreatic adenocarcinoma: The French SFRO-FFCD 97-04 Phase II trial. Int J Radiat Oncol Biol Phys 65: 1471-1478.

73. Talamonti MS, Small W, Jr., Mulcahy MF, Wayne JD, Attaluri V, et al. (2006) A multi-institutional phase II trial of preoperative full-dose gemcitabine and concurrent radiation for patients with potentially resectable pancreatic carcinoma. Ann Surg Oncol 13: 150-158.

74. White RR, Kattan MW, Haney JC, Clary BM, Pappas TN, et al. (2006) Evaluation of preoperative therapy for pancreatic cancer using a prognostic nomogram. Ann Surg Oncol 13: 1485-1492.

75. Wilkowski R, Thoma M, Bruns C, Wagner A, Heinemann V (2006) Chemoradiotherapy with gemcitabine and continuous 5-FU in patients with primary inoperable pancreatic cancer. Jop 7: 349-360.

76. Desai SP, Ben-Josef E, Normolle DP, Francis IR, Greenson JK, et al. (2007) Phase I study of oxaliplatin, full-dose gemcitabine, and concurrent radiation therapy in pancreatic cancer. J Clin Oncol 25: 4587-4592.

77. Fogelman DR, Schreibman S, Sherman W, Siegel AB, Ennis R, et al. (2007) Neoadjuvant GTX and radiation for unresectable pancreatic cancer: A prospective phase II trial. Gastrointestinal Cancers Symposium.

78. Macchia G, Valentini V, Mattiucci GC, Mantini G, Alfieri S, et al. (2007) Preoperative chemoradiation and intra-operative radiotherapy for pancreatic carcinoma. Tumori 93: 53-60.

79. Nakamori S, Kashiwazaki M, Tsujinaka T, Miyamoto A, Takeda Y, et al. (2007) Phase I study of neoadjuvant chemoradiation with gemcitabine and accelerated hyperfractionated radiation for potentially resectable pancreatic cancer. Gastrointestinal Cancers Symposium.

80. Palmer DH, Stocken DD, Hewitt H, Markham CE, Hassan AB, et al. (2007) A randomized phase 2 trial of neoadjuvant chemotherapy in resectable pancreatic cancer: gemcitabine alone versus gemcitabine combined with cisplatin. Ann Surg Oncol 14: 2088-2096.

81. Takamori H, Kanemitsu K, Chikamoto A, Ishiko T, Beppu T, et al. (2007) A novel multimodality treatment for resectable pancreatic cancer (PC). J Clin Oncol 25: 4630.

82. Vento P, Mustonen H, Joensuu T, Karkkainen P, Kivilaakso E, et al. (2007) Impact of preoperative chemoradiotherapy on survival in patients with resectable pancreatic cancer. World J Gastroenterol 13: 2945-2951.

83. Yamazaki H, Nishiyama K, Koizumi M, Tanaka E, Ioka T, et al. (2007) Concurrent chemoradiotherapy for advanced pancreatic cancer: 1,000 mg/m2 gemcitabine can be administered using limited-field radiotherapy. Strahlenther Onkol 183: 301-306.

84. Allendorf JD, Lauerman M, Bill A, DiGiorgi M, Goetz N, et al. (2008) Neoadjuvant chemotherapy and radiation for patients with locally unresectable pancreatic adenocarcinoma: feasibility, efficacy, and survival. J Gastrointest Surg 12: 91-100.

85. Brown KM, Siripurapu V, Davidson M, Cohen SJ, Konski A, et al. (2008) Chemoradiation followed by chemotherapy before resection for borderline pancreatic adenocarcinoma. Am J Surg 195: 318-321.

86. Evans DB, Varadhachary GR, Crane CH, Sun CC, Lee JE, et al. (2008) Preoperative gemcitabine-based chemoradiation for patients with resectable adenocarcinoma of the pancreatic head. J Clin Oncol 26: 3496-3502.

87. Golcher H, Brunner T, Grabenbauer G, Merkel S, Papadopoulos T, et al. (2008) Preoperative chemoradiation in adenocarcinoma of the pancreas. A single centre experience advocating a new treatment strategy. Eur J Surg Oncol 34: 756-764.

88. Greer SE, Pipas JM, Sutton JE, Zaki BI, Tsapakos M, et al. (2008) Effect of neoadjuvant therapy on local recurrence after resection of pancreatic adenocarcinoma. J Am Coll Surg 206: 451-457.

89. Heinrich S, Pestalozzi BC, Schafer M, Weber A, Bauerfeind P, et al. (2008) Prospective phase II trial of neoadjuvant chemotherapy with gemcitabine and cisplatin for resectable adenocarcinoma of the pancreatic head. J Clin Oncol 26: 2526-2531.

90. Le Scodan R, Mornex F, Partensky C, Mercier C, Valette PJ, et al. (2008) Histopathological response to preoperative chemoradiation for resectable pancreatic adenocarcinoma: the French Phase II FFCD 9704-SFRO Trial. Am J Clin Oncol 31: 545-552.

91. Lind PA, Isaksson B, Almstrom M, Johnsson A, Albiin N, et al. (2008) Efficacy of preoperative radiochemotherapy in patients with locally advanced pancreatic carcinoma. Acta Oncol 47: 413-420.

92. Marti JL, Hochster HS, Hiotis SP, Donahue B, Ryan T, et al. (2008) Phase I/II trial of induction chemotherapy followed by concurrent chemoradiotherapy and surgery for locoregionally advanced pancreatic cancer. Ann Surg Oncol 15: 3521-3531.

93. Small W, Mulcahy M, Benson A, Gold S, Rademaker F, et al. (2008) A phase II trial of weekly gemcitabine and bevacizumab in combination with abdominal radiation therapy in patients with localized pancreatic cancer. Gastrointestinal Cancers Symposium.

94. Stitzenberg KB, Watson JC, Roberts A, Kagan SA, Cohen SJ, et al. (2008) Survival after pancreatectomy with major arterial resection and reconstruction. Ann Surg Oncol 15: 1399-1406.

95. Takai S, Satoi S, Yanagimoto H, Toyokawa H, Takahashi K, et al. (2008) Neoadjuvant chemoradiation in patients with potentially resectable pancreatic cancer. Pancreas 36: e26-32.

96. Varadhachary GR, Wolff RA, Crane CH, Sun CC, Lee JE, et al. (2008) Preoperative gemcitabine and cisplatin followed by gemcitabine-based chemoradiation for resectable adenocarcinoma of the pancreatic head. J Clin Oncol 26: 3487-3495.

97. Bjerregaard JK, Mortensen MB, Jensen HA, Fristrup C, Svolgaard B, et al. (2009) Long-term results of concurrent radiotherapy and UFT in patients with locally advanced pancreatic cancer. Radiother Oncol.

98. Cardenes HR, Chiorean EG, Perkins S, DeWitt J, Schmidt M, et al. (2009) Long-term follow-up of a pilot study using neoadjuvant gemcitabine, erlotinib and hypofractionated radiation therapy for potentially resectable pancreatic cancer. Gastrointestinal Cancers Symposium.

99. Chaudhary UB, Gudena V, Cole S, O'Brien P, Montero AJ, et al. (2009) Preliminary results of a phase II neoadjuvant trial with gemcitabine/oxaliplatin and cetuximab followed by surgery or concurrent intensity modulated radiation therapy (IMRT) with capecitabine for patients with borderline resectable and unresectable nonmetastatic pancreatic cancer. Gastrointestinal Cancers Symposium.

100. Chen J, Li D, Killary AM, Sen S, Amos CI, et al. (2009) Polymorphisms of p16, p27, p73, and MDM2 modulate response and survival of pancreatic cancer patients treated with preoperative chemoradiation. Ann Surg Oncol 16: 431-439.

101. Choi M, Heilbrun LK, Venkatramanamoorthy R, Lawhorn-Crews JM, Zalupski MM, et al. (2009) Using 18F-Fluorodeoxyglucose Positron Emission Tomography to Monitor Clinical Outcomes in Patients Treated With Neoadjuvant Chemo-Radiotherapy for Locally Advanced Pancreatic Cancer. Am J Clin Oncol.

102. Kim YE, Park MS, Hong HS, Kang CM, Choi JY, et al. (2009) Effects of neoadjuvant combined chemotherapy and radiation therapy on the CT evaluation of resectability and staging in patients with pancreatic head cancer. Radiology 250: 758-765.

103. Laurent S, Monsaert E, Boterberg T, Demols A, Borbath I, et al. (2009) Feasibility of radiotherapy with concomitant gemcitabine and oxaliplatin in locally advanced pancreatic cancer and distal cholangiocarcinoma: a prospective dose finding phase I-II study. Ann Oncol.

104. Masui T, Doi R, Kawaguchi Y, Koizumi M, Kida A, et al. (2009) Gemcitabine and S-1 combined neoadjuvant chemotherapy for patients with locally advanced pancreatic cancer. Gastrointestinal Cancers Symposium.

105. Maximous DW, Abdel-Wanis ME, El-Sayed MI, Abd-Elsayed AA (2009) Preoperative gemcitabine based chemo-radiotherapy in locally advanced non metastatic pancreatic adenocarcinoma. Int Arch Med 2: 7.

106. Ohigashi H, Ishikawa O, Eguchi H, Takahashi H, Gotoh K, et al. (2009) Feasibility and efficacy of combination therapy with preoperative full-dose gemcitabine, concurrent three-dimensional conformal radiation, surgery, and postoperative liver perfusion chemotherapy for T3-pancreatic cancer. Ann Surg 250: 88-95.

107. Rifkind J, Barth Jr R, Zaki B, Ripple G, Tsapakos M, et al. (2009) Neoadjuvant chemoradiotherapy for pancreatic cancer: The Dartmouth experience. Gastrointestinal Cancers Symposium.

108. Satoi S, Yanagimoto H, Toyokawa H, Takahashi K, Matsui Y, et al. (2009) Surgical results after preoperative chemoradiation therapy for patients with pancreatic cancer. Pancreas 38: 282-288.

109. Stokes JB, Walters DM, Nolan NJ, Stelow EB, Rich TA, et al. (2009) Outcome following neoadjuvant therapy for borderline resectable pancreatic cancer. Gastrointestinal Cancers Symposium.

110. Tinkl D, Grabenbauer GG, Golcher H, Meyer T, Papadopoulos T, et al. (2009) Downstaging of pancreatic carcinoma after neoadjuvant chemoradiation. Strahlenther Onkol 185: 557-566.

111. Turrini O, Viret F, Moureau-Zabotto L, Guiramand J, Moutardier V, et al. (2009) Neoadjuvant 5 fluorouracil-cisplatin chemoradiation effect on survival in patients with resectable pancreatic head adenocarcinoma: a ten-year single institution experience. Oncology 76: 413-419.
