# Supplementary material for: Preoperative/Neoadjuvant Therapy in Pancreatic Cancer: A Systematic Review and Meta-analysis of Response and Resection Percentages
Source: PLoS Med. 2010 Apr 20;7(4):e1000267. doi: 10.1371/journal.pmed.1000267 (PMC2857873; doi:10.1371/journal.pmed.1000267)
Supplement: Table S2 — Currently ongoing/recruiting trials for resectable or non-resectable but non-metastatic PDAC. (0.07 MB DOC) [file pmed.1000267.s003.doc]

**Table S2**: Currently ongoing/recruiting (as of December 1, 2009) trials for resectable or non-resectable but non-metastatic PDAC

|  | Main trial ID | Phase | Title |
| --- | --- | --- | --- |
| **Neoadjuvant trials for resectable PDAC** | NCT00262951 | Phase II | A Phase II Pilot Study of Multi-Agent Neo-Adjuvant Chemoradiation in Patients With Locally Advanced Pancreatic Adenocarcinoma |
|  | NCT00426738 | Phase II | A Multi-Institutional Phase II Study of Neoadjuvant Gemcitabine and Oxaliplatin With Radiation Therapy in Patients With Pancreatic Cancer |
|  | NCT00438256 | Phase I/II | Phase I/II of Neoadjuvant Accelerated Short Course Radiation Therapy With Proton Beam and Capecitabine for Resectable Pancreatic Cancer |
|  | NCT00456599 | Phase II | A Multi-Institutional Phase II Study of Neoadjuvant Gemcitabine and Oxaliplatin With Radiation Therapy in Patients With Pancreatic Cancer |
|  | NCT00536874 | Phase II | A Phase II Study of Neoadjuvant Gemcitabine and Oxaliplatin in Patients With Potentially Resectable Previously Untreated Pancreatic Adenocarcinoma |
|  | NCT00557492 | Phase II | Phase II Study of the Anti-Vascular Endothelial Growth Factor (a-VEGF) Monoclonal Antibody Bevacizumab in Combination With Fixed Dose Rate (FDR) Gemcitabine and Rapid-Fractionation Radiotherapy in the Pre-Operative Treatment of Potentially- Resectable Pancreatic Adenocarcinoma |
|  | NCT00602602 | Phase II | A Phase II Study of Gemcitabine, Oxaliplatin and Bevacizumab Followed by 5-Fluorouracil, Oxaliplatin, Bevacizumab and Radiotherapy in Patients With Locally Advanced Pancreatic Cancer |
|  | NCT00609336 | Phase II | A Phase II Study Induction Chemotherapy, Neoadjuvant Chemoradiotherapy, Surgical Resection and Adjuvant Chemotherapy for Patients With Locally Advanced, Resectable Pancreatic Adenocarcinoma |
|  | NCT00614653 | Phase I | Phase I Trial of Preoperative Radiotherapy With Concurrent Bevacizumab, Erlotinib and Capecitabine for Locally Advanced Pancreatic Cancer |
|  | NCT00705393 | Phase I | A Phase 1 Study of Hypofractionated Stereotactic Radiotherapy and Concurrent HIV Protease Inhibitor Nelfinavir as Part of a Neoadjuvant Regimen in Patients With Locally Advanced Pancreatic Cancer |
|  | NCT00727441 | Phase I | A Randomized Three-Arm Neoadjuvant and Adjuvant Feasibility and Toxicity Study of a GM-CSF Secreting Allogeneic Pancreatic Cancer Vaccine Administered Either Alone or in Combination With Either a Single Intravenous Dose or Daily Metronomic Oral Doses of Cyclophosphamide for the Treatment of Patients With Surgically Resected Adenocarcinoma of the Pancreas |
|  | NCT00733746 | Phase II | A Phase II Study of Preoperative Gemcitabine and Erlotinib Plus Pancreatectomy and Postoperative Gemcitabine and Erlotinib for Patients With Operable Pancreatic Adenocarcinoma |
|  | NCT00763516 | Phase I | A Pilot Study Using Neoadjuvant Proton Beam Radiation Therapy and Chemotherapy for Marginally Resectable Carcinoma of the Pancreas |
|  | NCT00766636 | Phase II | A Randomized Phase II Study of Preoperative Chemotherapy (Gemcitabine and Erlotinib) With or Without Radiation Therapy for Patients With Resectable Adenocarcinoma of the Pancreas |
|  | NCT00833859 | Phase II | A Phase 2 Study of GTX-SRS: Neoadjuvant Gemcitabine, Docetaxel, and Capecitabine in Combination With Stereotactic Radiosurgery for Borderline Resectable Pancreatic Cancer |
|  | NCT00869258 | Phase II | Phase II Study for Inoperable Non-Metastatic Pancreatic Cancer (Stage IVA) With Neoadjuvant Gemzar, Taxotere and Xeloda (GTX), and Radiation With Gemzar |
|  | NCT00889187 | Phase I/II | Phase I/II Study of Neoadjuvant Accelerated Short Course Radiation Therapy With Photons and Capecitabine for Resectable Pancreatic Cancer |

|  | Main trial ID | Phase | Title |
| --- | --- | --- | --- |
| **Trials for non-resectable but non-metastatic PDAC, i.e. potentially neoadjuvant** | ISRCTN  96169987 | Phase II | A multi-centre randomised phase II study of induction chemotherapy followed by gemcitabine or capecitabine based chemoradiotherapy (CRT) for locally advanced non-metastatic pancreatic cancer |
|  | NCT00051467 | Phase II/III | A Randomized, Phase II/III, Study of TNFerade™ Biologic With 5-FU and Radiation Therapy for First-line Treatment of Unresectable Locally Advanced Pancreatic Cancer |
|  | NCT00149578 | Phase II | A Phase II Study of Induction Chemotherapy Followed by Concurrent Chemotherapy With Radiotherapy in Locally Advanced Pancreatic Cancer |
|  | NCT00226746 | Phase II | A Multicenter Phase II Trial of Weekly Gemcitabine, Paclitaxel, and Hyperfractionated External Irradiation (63.80 GY) for Locally Advanced Pancreatic Cancer |
|  | NCT00288093 | Phase I | A Phase I Study of Triapine® in Combination With Radiation Therapy in Locally Advanced Pancreas Cancer |
|  | NCT00304135 | Phase II/III | Randomized Phase II-III Study of Chemoradiation With Fluorouracil and Cisplatin Versus Chemotherapy (Gemcitabine/Oxaliplatin) in Non Resectable But Non Metastatic Cancer of the Biliary Tract |
|  | NCT00346281 | Phase II | A Phase IIa, Safety Study of the Active Implantable (Radiological) Medical Device 32P BioSilicon, Administered Intratumourally to Patients With Advanced, Unresectable Pancreatic Cancer, in Addition to Standard IV Gemcitabine Chemotherapy |
|  | NCT00375310 | Phase I | Phase I Study of Gemcitabine With Novel RAF Kinase-Vascular Endothelial Growth Factor Receptor Inhibitor Sorafenib (BAY 43-9006) and Radiotherapy in Patients With Locally Advanced Unresectable Pancreatic Adenocarcinoma |
|  | NCT00415454 | Phase I | Phase I Study Combining Suicide Gene Therapy With Chemoradiotherapy in the Treatment of Non-Metastatic Pancreatic Adenocarcinoma |
|  | NCT00424827 | Phase II | A Phase II Trial of Cetuximab, Gemcitabine, 5-Fuorouracil and Radiation Therapy in Locally Advanced Non-metastatic Pancreatic Adenocarcinoma |
|  | NCT00565487 | Phase I | Phase I Study of Combination of Capecitabine and Erlotinib Concurrent With Radiotherapy in Patients With Non-Operable Locally Advanced Pancreatic Cancer |
|  | NCT00593866 | Phase I/II | A Phase I/II Radiation Dose-Escalation Study of Intensity-Modulated Radiotherapy (IMRT) With Concurrent Gemcitabine in Patients With Unresectable Pancreatic Cancer |
|  | NCT00599833 | Phase II | Locally Advanced Pancreatic Cancer: Phase II Study of Cetuximab and 3-D Conformal Image Guided Radiotherapy (PACER) |
|  | NCT00601627 | Phase II | Phase II Study of Panitumumab, Chemotherapy, and External Beam Radiation in Patients With Locally Advanced Pancreatic Cancer |
|  | NCT00634725 | Phase III | Randomized Multicenter Phase III Study in Patients With Locally Advanced Adenocarcinoma of the Pancreas: Gemcitabine With or Without Chemoradiotherapy and With or Without Erlotinib. Intergroup Study |
|  | NCT00658840 | Phase II | A Phase II Study of Concurrent Chemo-Radiotherapy With Capecitabine for Unresectable Locally Advanced Pancreatic Carcinoma |
|  | NCT00685763 | Phase I | A Study Using Photon/Proton Beam Radiation Therapy and Chemotherapy for Unresectable Carcinoma of the Pancreas |
|  | NCT00711997 | Phase I/II | Phase 1/2a, Dose-Escalation, Safety, Pharmacokinetic, and Preliminary Efficacy Study of Intratumoral Administration of DTA-H19 in Patients With Unresectable Pancreatic Cancer |
|  | NCT00735306 | Phase I/II | A Phase I/II Trial of Radiation, Avastin and Tarceva for Resectable or Locally Advanced Pancreatic Adenocarcinoma |
|  | NCT00831493 | Phase I/II | Phase I/II Trial of Vorinostat and Radiation Therapy in Patients With Locally Advanced Pancreatic Cancer |
|  | NCT00878657 | Phase I/II | Phase I/II Radiotherapy Dose Escalation Study in Locally Advanced Pancreatic Cancer, Using a Simultaneous Intensity Modulated Boost With Concurrent Gemcitabine |
|  | NCT00948688 | Phase I/II | Phase 1/2 Study of Vorinostat in Combination With Radiation Therapy and Infusional 5-FU in Patients With Locally Advanced Adenocarcinoma of the Pancreas |
|  | NCT00983268 | Phase I | Phase I Trial of Chemoradiation With Capecitabine and Vorinostat in Pancreatic Cancer |
