# Supplementary material for: Preoperative/Neoadjuvant Therapy in Pancreatic Cancer: A Systematic Review and Meta-analysis of Response and Resection Percentages
Source: PLoS Med. 2010 Apr 20;7(4):e1000267. doi: 10.1371/journal.pmed.1000267 (PMC2857873; doi:10.1371/journal.pmed.1000267)
Supplement: Text S2 — Review protocol. (0.04 MB DOC) [file pmed.1000267.s005.doc]

**Review protocol**

**Primary database:** MEDLINE, EMBASE, Cochrane Central Register of Controlled Trials

**Search terms:**

(pancreas OR pancreatic) AND

(cancer OR carcinoma) AND

(neoadjuvant OR preoperative) AND

(radiation OR chemoradiation OR chemotherapy)

**Search terms for trials:**

[(pancreas OR pancreatic) AND

(cancer OR carcinoma) AND

(neoadjuvant OR preoperative)]

OR

[(pancreas OR pancreatic) AND

(cancer OR carcinoma) AND

(non-metastatic OR nonmetastatic) AND

(unresectable OR non-resectable OR locally advanced)]

**Additional databases:**

Proceedings of the Gastrointestinal Cancers Symposium and ASCO Annual Meeting

ISRCTN Register, Action Medical Research, Leukaemia Research Fund

Medical Research Council (UK), National Health Service Research and Development Health Technology Assessment Programme (HTA), National Institutes of Health (www.ClinicalTrials.gov), The Wellcome Trust, UK Clinical Trials Gateway

WHO International Clinical Trials Registry Platform (www.who.int/ictrp) including Australian New Zealand Clinical Trials Registry (ANZCTR), Chinese Clinical Trial Register (ChiCTR), Clinical Trials Registry - India (CTRI), German Clinical Trials Register (DRKS), Iranian Registry of Clinical Trials (IRCT), Sri Lanka Clinical Trials Registry (SLCTR), and The Netherlands National Trial Register (NTR)

**Additional searching:**

Reference review (any article/trial pulled for possible inclusion)

The search is done independently by both SG and JK. Any article/trial felt suitable by either reviewer is included for closer examination.

**Inclusion/exclusion**

**Inclusion criteria:**

1) Pancreatic and/or periampullary cancer

2) Neoadjuvant (preoperative) radiochemotherapy or radiotherapy or chemotherapy

3) Re-staging following therapy

4) Surgical exploration/re-exploration/resection in selected patients

**Exclusion criteria:**

1) No surgical exploration/re-exploration/resection following therapy

2) Only immunotherapy as preoperative therapy

3) Only genetic prediction analysis

4) Only radiographical sensitivity/specificity analysis

5) Identical patient cohorts

6) Case reports

Study inclusion/exclusion is completed independently (SG & JK). Results are reviewed (SG) and any disagreement is recorded. Results are discussed (SG & JK) to reach consensus.

**Data abstraction form**

**Identification of data abstractor:**

1. Initials of abstractor

**Identification of study:**

2. Record the first authors’ last name, initials

3. Record the authors’ institution

4. Record the journal name

5. Record the year of publication

6. Record the volume number

7. Record the page numbers

8. Publication status

- 1. Full study
  2. Abstract

# Characteristics of study:

9. Study period

10. Retrospective/prospective analysis

11. Trial phase

a. phase I

b. phase I/II

c. phase II

12. Cohort study/case series

13. Tumor entity

a. pancreatic cancer

b. periampullary cancer

14. Initial tumor assessment

a. resectable

b. non-resectable (borderline/unresectable)

15. Resectability criteria: NCCN, defined criteria, not clearly defined/not stated

16. Histological tumor verification prior therapy

a. yes

b. no

17. If chemotherapy was applied: exact drug regimen

18. If radiotherapy was applied: exact doses/protocol

19. Grade 3/4 toxicity of neoadjuvant therapy

20. If intraoperative radiotherapy was applied: exact dosage

21. Record of patient age

**Outcome characteristics:**

22. Number of patients treated neoadjuvant that were re-staged

23. Number of patients treated neoadjuvant that were explored

24. Number of patients treated neoadjuvant that were resected

25. Response evaluation: RECIST, defined, not clearly defined/not stated

26. Response evaluation

a. radiographical

b. histological

c. both

d. none

27. Record of response

a. complete response

b. partial response

c. stable disease

d. progressive disease

28. Record of R0 resection rate

29. Record of local recurrence rate

30. Survival estimates (for all, resected, non-resected patients as applicable)

a. 1 year survival

b. 2 year survival

c. 3 year survival

d. 5 year survival

e. median survival

31. Record of postoperative morbidity

32. Record of postoperative mortality

Data abstraction is completed independently (SG & JK). Results are reviewed (SG) and any disagreement is recorded. Results are discussed (SG & JK) to reach consensus.

Statistical participation (TS) regarding potential combination of data and analysis.
